# Advances in the Search for SARS-CoV-2 M^pro^ and PL^pro^ Inhibitors

**DOI:** 10.3390/pathogens13100825

**Published:** 2024-09-24

**Authors:** Marcel Arruda Diogo, Augusto Gomes Teixeira Cabral, Renata Barbosa de Oliveira

**Affiliations:** Departamento de Produtos Farmacêuticos, Faculdade de Farmácia, Universidade Federal de Minas Gerais, Belo Horizonte 31270-901, Brazil; marcel.arruda@farmacia.ufjf.br (M.A.D.); augusto.bhzt@gmail.com (A.G.T.C.)

**Keywords:** cysteine protease, M^pro^, PL^pro^, inhibitors, SARS-CoV-2

## Abstract

SARS-CoV-2 is a spherical, positive-sense, single-stranded RNA virus with a large genome, responsible for encoding both structural proteins, vital for the viral particle’s architecture, and non-structural proteins, critical for the virus’s replication cycle. Among the non-structural proteins, two cysteine proteases emerge as promising molecular targets for the design of new antiviral compounds. The main protease (M^pro^) is a homodimeric enzyme that plays a pivotal role in the formation of the viral replication–transcription complex, associated with the papain-like protease (PL^pro^), a cysteine protease that modulates host immune signaling by reversing post-translational modifications of ubiquitin and interferon-stimulated gene 15 (ISG15) in host cells. Due to the importance of these molecular targets for the design and development of novel anti-SARS-CoV-2 drugs, the purpose of this review is to address aspects related to the structure, mechanism of action and strategies for the design of inhibitors capable of targeting the M^pro^ and PL^pro^. Examples of covalent and non-covalent inhibitors that are currently being evaluated in preclinical and clinical studies or already approved for therapy will be also discussed to show the advances in medicinal chemistry in the search for new molecules to treat COVID-19.

## 1. Introduction

**Figure 1:**
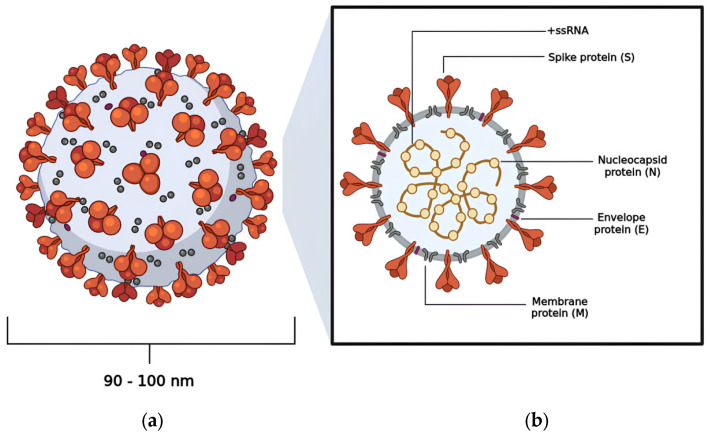
The viral structure exhibited by SARS-CoV-2: (**a**) the spherical shape adopted by SARS-CoV-2; (**b**) the distribution of the virus’s structural proteins—nucleocapsid protein (N), membrane protein (M), envelope protein (E), and spike protein (S).

Coronavirus Disease 2019 (COVID-19) is a highly contagious illness caused by the betacoronavirus known as SARS-CoV-2 [1,2]. As of the latest reports, there have been over 775,867,547 (19 August 2024) confirmed cases worldwide, resulting in more than 6,881,955 deaths [3]. Morphological analysis obtained by cryogenic electron microscopy (Cryo-EM) reveals that this virus typically displays a spherical shape, with a diameter ranging from 90 to 100 nm [4,5]. Notably, the virus features a large homotrimeric glycoprotein (~600 kDa) protruding from its surface, which plays a crucial role in mediating interactions with host cells during the infection process, similar to other viruses within the same genus (Figure 1) [6,7].

**Figure 2:**
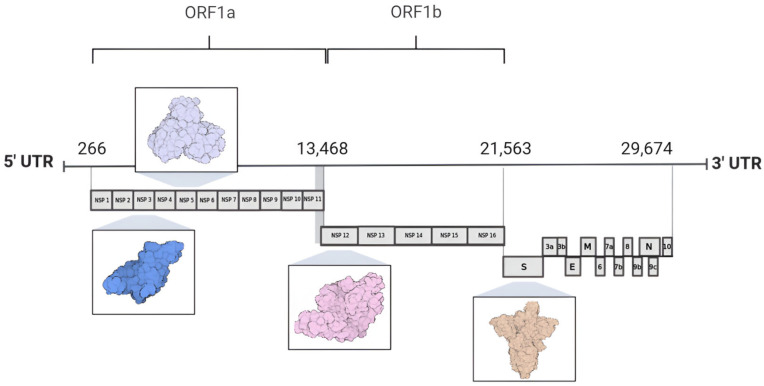
Genome organization of SARS-CoV-2. The 5′ region of the SARS-CoV-2 genome comprises open reading frames ORF1a and ORF1b, which encode 16 non-structural proteins. In contrast, the 3′ region of the genome encodes four proteins involved in the assembly of the viral particle structure, alongside nine accessory proteins. Highlighted among these are proteins used as molecular targets for drug design and development: nsp3 (PL^pro^ PDB ID 7NFV), nsp5 (M^pro^ PDB ID 7BB2), nsp12 (RdRp PDB ID 7BV1), and the S protein (spike protein PDB ID 7DK3).

The SARS-CoV-2 genome consists of a single-stranded, positive-sense RNA molecule (+ssRNA) that is approximately 30 kb in length and encodes 29 proteins, including non-structural proteins (nsps 1–16), structural proteins (spike protein, envelope, membrane, and nucleocapsid), and accessory proteins [6,8]. At the 5′ end of the genome lie two substantial open reading frames (ORFs), designated as ORF1a and ORF1b, encompassing two thirds of the total genome size [9,10]. These ORFs are subsequently translated into polyprotein 1a (pp1a) and polyprotein 1ab (pp1ab) by the host cell ribosomes, resulting in the synthesis of non-structural proteins [11,12]. In contrast, the 3′ end is associated with the translation of proteins crucial for the viral particle architecture, which consist of the nucleocapsid protein (N), membrane protein (M), envelope protein (E), and spike protein (S), alongside nine accessory proteins [4,13,14]. The accessory protein composition varies significantly among viral species and includes ORF3a, ORF3b, ORF6, ORF7a, ORF7b, ORF8, ORF9b, ORF9c, and ORF10 (Figure 2) [7,8,15].

### SARS-CoV-2 Life Cycle

**Figure 3:**
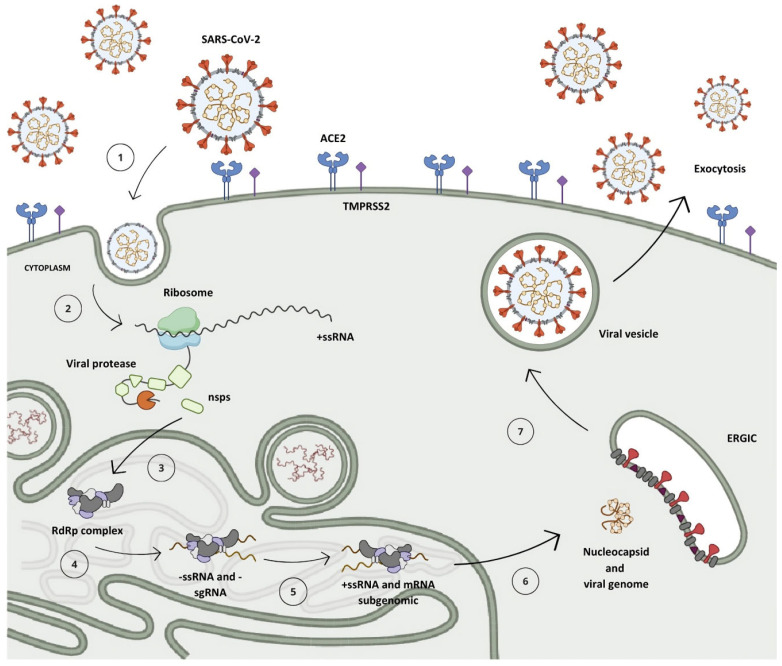
The replication mechanism of the SARS-CoV-2 virus. (1) Virus interaction and entry into the host cell. (2) Polyprotein translation. (3) Cleavage of polyproteins by PL^pro^ (nsps 1–3) and M^pro^ (nsps 4–16) enzymes and assembly of viral translation and replication complex. (4) Synthesis of negative-sense genomic and subgenomic RNA strands. (5) Synthesis of positive-sense RNA strands and subgenomic messenger RNA. (6) Translation of subgenomic messenger RNA into structural and accessory proteins and assembly of new viral particle. (7) Viral particle formation and incorporation into vesicles. (8) Release of new viral particle by exocytosis.

After penetrating the human organism, the receptor-binding domain (RBD) situated in the S protein, anchored on the SARS-CoV-2 surface, engages with the angiotensin-converting enzyme 2 (ACE2) found in host cells, predominantly within type II pneumocytes (Figure 3, step 1) [5,16,17]. This interaction initiates the cascade of events leading to viral infection and replication [11].

Within the cellular environment, the open reading frames ORF1a and ORF1b of the genomic RNA (gRNA) undergo translation into polyproteins pp1a and pp1ab via a mechanism known as the -1 programmed ribosomal frameshift, which enables the reading of the ORF1b region (Figure 3, step 2) [18,19]. During the synthesis of pp1a, the non-structural protein 1 (nsp1) situated at the *N*-terminal segment of the polyprotein undergoes proteolytic cleavage mediated by nsp3 (PL^pro^), releasing it from the chain (Figure 3, step 3) [20,21]. This process enables its interaction with the ribosome’s 40S subunit, consequently inhibiting protein synthesis and initiating the degradation of the host mRNA [8,22]. Subsequent to this, the nsp3 protease cleaves nsp2 prior to self-cleaving, dissociating from the chain and aiding in viral evasion processes by modulating the host’s immune response through the reversal of post-translational modifications of ubiquitin and interferon-stimulated gene 15 (ISG15) [19,23,24].

A second protease, known as the M^pro^, is synthesized from the ORF1 frame integrated into the pp1a chain, referring to non-structural protein 5 (nsp5), which undergoes autocleavage, releasing nsp4 and instigating a cascade of proteolytic cleavages at specific sites among the remaining 11 non-structural proteins, resulting in the assembly of the viral replication–transcription complex (Figure 3, step 3) [9,25].

Following the replication–transcription complex’s assembly, full-length strands of negative-sense RNA are synthesized and serve as templates for the generation of a new positive-sense RNA (+ssRNA) molecule, which can either be integrated into a new viral particle or translated into non-structural proteins [11,15]. Furthermore, due to a mechanism of discontinuous transcription, subgenomic negative-sense RNA (sgRNA) strands are synthesized, leading to the production of subgenomic positive-sense messenger RNA (sg-mRNA) molecules, which are responsible for the production of structural and accessory proteins related to the architecture of SARS-CoV-2 (Figure 3, steps 4 and 5) [26,27,28,29].

Upon completion of genomic RNA replication, the genetic material synthesized within the replication organelles is secreted into the cytoplasm of the host cell via a transmembrane pore, where it binds to the nucleocapsid protein in a helical conformation [18,30,31]. Following this, structural proteins anchored in the SARS-CoV-2 membrane are translated in the endoplasmic reticulum and then shuttled to the intermediate compartment situated between the Golgi complex and the endoplasmic reticulum (ERGIC, endoplasmic reticulum–Golgi intermediate compartment), where they interact with the genetic material encased by the N protein, resulting in the assembly of a new virion (Figure 3, step 6) [11,27,32]. Then, these virions are subsequently encapsulated within lysosomal vesicles and released from the infected cell through the process of exocytosis (Figure 3, steps 7 and 8) [33,34].

## 2. Cysteine Protease as a Target for the Discovery of New Drugs

Cysteine proteases are enzymes that occur in a wide variety of biological systems, being found in eukaryotic organisms such as plant, fungal, and animal cells, as well as in prokaryotic organisms such as bacterial cells and viral particles [35,36]. Phylogenetic analyses based on the evolutionary origin of the enzyme and the composition of the amino acid residues present in its structure reveal that cysteine proteases are organized into 15 distinct clades named CA, CD, CE, CF, CL, CM, CN, CO, CP, CQ, CR, PA, PB, PC, and PD, which encompass over 101 families distributed among the clades (C1–C124) [37,38,39].

The catalytic mechanism presented by cysteine proteases starts with the formation of the nucleophilic thiolate ion through the deprotonation of a cysteine residue located in the enzyme’s catalytic cavity, promoted by the nitrogen atom of the imidazole ring on the side chain of a histidine residue [40,41]. This catalytic dyad is commonly observed in cysteine proteases belonging to the PA clan, such as the main protease (M^pro^) of SARS-CoV-2, frequently employed in the study of new drug candidates for COVID-19 treatment [38,42,43]. Conversely, enzymes from the CA clan, such as the papain-like protease (PL^pro^) of SARS-CoV-2, feature a catalytic triad, which includes not only cysteine and histidine residues but also an aspartate residue in its catalytic cavity, sometimes replaced by asparagine in other organisms [35,37,44,45].

Given the critical role played by the cysteine proteases PL^pro^ and M^pro^, the significant similarity between their amino acid residue sequences, their counterparts present in SARS-CoV (identities of 83% and 96%, respectively), and the lack of similarity with human proteins, these enzymes are considered crucial molecular targets for the rational design and development of new broad-spectrum antiviral drugs [10,43,46,47].

### 2.1. Main Protease (M^pro^): Structure, Function, and Mechanism of Catalysis

The M^pro^ enzyme (EC 3.4.22.69), also known as 3-chymotrypsin-like protease (3CLpro), is a cysteine protease with high conservation among Coronaviridae family members and plays a pivotal role in cleaving the polyproteins pp1a and pp1ab at multiple sites, leading to the formation of the viral replication–transcription complex [48,49]. Structural analyses have shown that the protease consists of two identical polypeptide chains, each approximately 33.8 kDa in size, composed of 306 amino acid residues, which interact to form a catalytically active homodimer [50,51].

**Figure 4:**
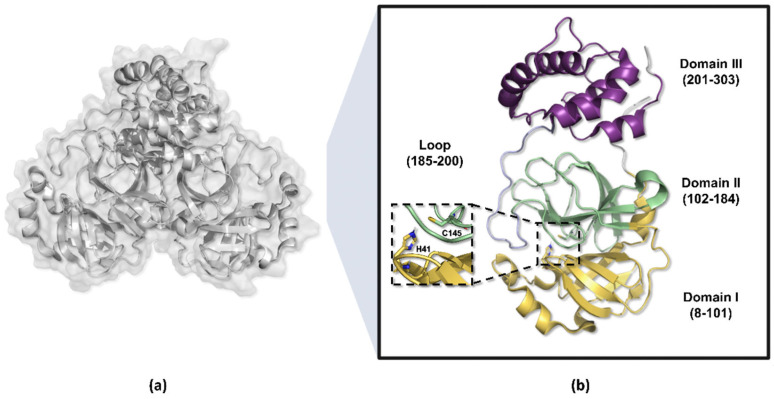
Three-dimensional structure of the SARS-CoV-2 M^pro^ enzyme. (**a**) Homodimeric structure of the M^pro^ enzyme (PDB ID 7BB2). (**b**) Monomer of the enzyme, weighing approximately 33.8 kDa, divided into three domains: domains I (yellow) and II (green) perform catalytic functions, while domain III (purple) is responsible for molecule dimerization.

The amino acid residues 8 to 101 in the M^pro^ enzyme structure are responsible for forming the protomer’s domain I, while residues 102 to 184 compose domain II, which adopts a barrel-shaped motif characterized by six antiparallel beta sheets that fold into a chymotrypsin-like configuration, harboring the catalytic dyad His41-Cys145 positioned between these two domains [52,53,54]. Conversely, residues 201 to 303 constitute the protomer’s domain III, organized into five alpha helices and responsible for dimerization, connected to domain II via a long loop composed of residues 185 to 200 (Figure 4) [49,55,56].

The M^pro^ enzyme’s dimerization is primarily coordinated by the formation of an ionic bond between the Arg04 and Glu290 residues, arranged in opposite chains, which is of paramount importance for the enzyme’s catalytic activity [53,57]. This configuration allows the amino group of the serine residue, located in the *N*-terminal portion of one protomer (*N*-finger), to position itself between domains I and II of the other chain [58,59]. This strategic alignment enables a hydrogen bond interaction with the Glu166 residue, thereby facilitating the formation of the S1 pocket within the substrate-binding site [52,60].

The catalytic cavity of the M^pro^ enzyme is characterized by four principal pockets, known as S1, S1′, S2, and S4, which accommodate distinct functional groups present in the substrate [51,53]. The S1 pocket demonstrates high specificity for glutamine residues or analogs mimicking its side chain, while S1′ accommodates the catalytic dyad comprising Cys145 and His41 residues [52,61,62]. Additionally, S2 exhibits notable plasticity, allowing adaptation to diverse lipophilic groups such as leucine and phenylalanine, a characteristic shared with the S4 pocket [55,61,63].

The cleavage site in the polyproteins, conventionally targeted by the M^pro^, is composed of -Leu-Gln↓Ser-Ala-Gly- residues, where peptide bond hydrolysis occurs between the Gln and Ser residues [51,57].

**Figure 5:**
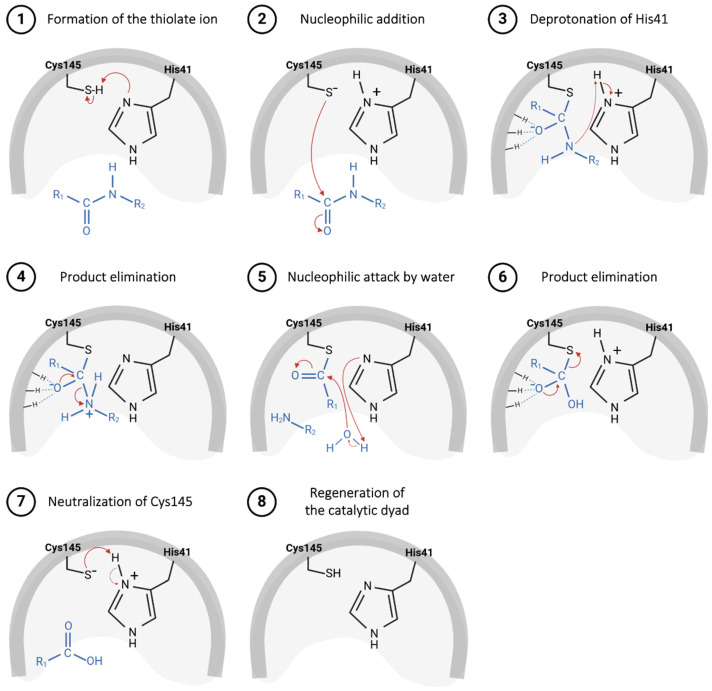
Proposed catalytic mechanism for SARS-CoV-2 M^pro^ enzyme.

The catalytic mechanism proposed for the M^pro^ starts with proton abstraction from the thiol moiety of the Cys145’s side chain, situated within domain II, promoted by the imidazole ring of His41, positioned in domain I (Figure 5, step 1) [43]. This proton transference event culminates in the formation of a highly reactive thiolate ion, which, through nucleophilic addition to the carbonyl carbon of the substrate’s peptide bond (Figure 5, step 2), yields a thio-hemiacetal intermediate, as represented in Figure 5, step 3 [64]. Then, His41 acts as an acid and donates a proton to the nitrogen of the intermediate moiety (Figure 5, step 3), leading to the release of the polypeptide chain, catalyzed by the restoration of the carbonyl π bond (Figure 5, step 4), thereby forming the acyl–enzyme complex [49]. Shortly after this, the carbon of the thioester group is attacked by a water molecule (Figure 5, step 5), leading again to the protonation of the His41 residue and the subsequent release of the second polypeptide chain (Figure 5, step 6), restoring the initial thiolate moiety, which will be neutralized by capturing the proton from His41 (Figure 5, steps 7 and 8) [65,66].

Based on this mechanism, a range of compounds containing an electrophilic group that could be attacked by a Cys residue have been developed as potential covalent inhibitors of the M^pro^.

### 2.2. Papain-like Protease (PL^pro^): Structure, Function, and Mechanism of Catalysis

The non-structural protein 3 (nsp3) is the largest protein encoded by the SARS-CoV-2 genome, composed of 1945 amino acid residues and responsible for the formation of multiple protein domains [67]. During viral infection, nsp3 plays a variety of crucial roles, including the biogenesis of double-membrane vesicles (DMVs) by forming a heterodimer with nsp4 and nsp6, the cleavage of the viral polyprotein to release nsp1, and the evasion of the host immune response [24,68,69]. Additionally, nsp3 acts as a membrane-anchored scaffold, facilitating the recruitment of both coronavirus and host proteins, as well as viral RNA, to initiate the assembly of replication–transcription complexes (RTCs) within infected cells [67].

**Figure 6:**
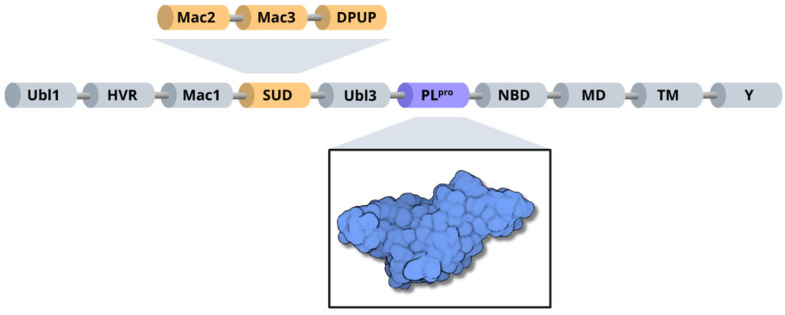
Organization of SARS-CoV-2 nsp3 domains, highlighting the PL^pro^ enzyme (blue) and the three subdomains of SARS-unique domain (orange): Mac2, Mac3 and DPUP.

Structurally, SARS-CoV-2 nsp3 can be subdivided into 10 distinct domains. Residues 1–108 form the ubiquitin-like domain 1 (Ubl1), followed by a hypervariable region (HVR) comprising residues 109–206 [10]. Next, the macrodomain I (Mac1) is formed by residues 207–386, and the subsequent “SARS-unique domain” (SUD) is divided into three subdomains: macrodomain II (Mac2); encompassing residues 387–548, macrodomain III (Mac3), consisting of residues 549–676; and the domain preceding Ubl2 and PL2pro (DPUP), formed by residues 677–745 [70,71]. Additionally, nsp3 contains the ubiquitin-like domain 2 (Ubl2), composed of residues 746–805, followed by the papain-like protease (PL^pro^), which includes residues 806–1058 [10]. The nucleic acid-binding domain (NBD) spans residues 1059–1200, the marker domain (MD) comprises residues 1201–1340, the transmembrane regions (TM) cover residues 1341–1567, and the Y domain is formed by residues 1568–1945 [10,67] (Figure 6).

The PL^pro^ (EC 3.4.22.46) is a cysteine protease similar to papain. It is composed of 315 residues with a size of 35.6 kDa, being responsible for cleaving nsp1 and nsp2, assisting in the phases of viral infection and replication [47,72,73]. Besides the cleavage mechanism, The PL^pro^ operates by modulating the host’s immunological signaling, through reversing the post-translational modifications of ubiquitin and of the interferon-stimulated gene 15 (ISG15) in the host’s cells. This gene is the main path used by SARS-CoV-2, resulting in the suppression of the innate immunity, making the PL^pro^ a key molecular target in the development of new antivirals dedicated to the treatment of COVID-19 [69,74,75].

**Figure 7:**
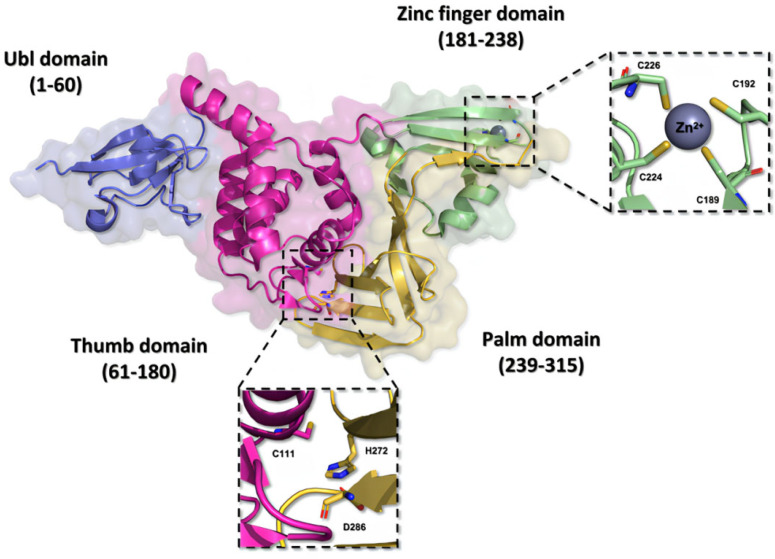
Three-dimensional structure of the SARS-CoV-2 PL^pro^ enzyme. The monomeric structure of the PL^pro^ enzyme (PDB ID 7NFV) features a catalytic triad composed of the residues Cys111, His272, and Asp286, located within the thumb and palm domains. The zinc finger domain hosts a zinc ion, stabilized by residues Cys189, Cys192, Cys224, and Cys226, contributing to the stability of the protein.

Structurally, the PL^pro^ is very similar to the human cellular deubiquitinases (DUBs), specifically ubiquitin-specific proteases 12 and 14 (USP12 and USP14), due to their organization in the thumb–palm–finger domains (Figure 7) [23,76,77].

The ubiquitin-like (Ubl) domain located in the *N*-terminal region of the PL^pro^ enzyme is formed by amino acid residues 1 to 60, organized into a structure comprising three beta sheets and one alpha helix, imparting conformational flexibility [73]. Moreover, the residues located between positions 61 and 180 in the polypeptide chain fold themselves into six alpha helices composing the thumb domain, which contains the Cys111 residue, a key component of the catalytic triad of the enzyme [76,78]. The zinc finger domain consists of the residues from 181 to 238, and it is organized in four beta sheets and two alpha helices. This domain contains a zinc ion, coordinated with four cysteine residues (Cys189, Cys192, Cys224, and Cys226) in a tetrahedral conformation, located in two protein loops, which is essential to the structural stability and to the catalytic properties of the protease [48,73]. On the other hand, the *C*-terminal portion of the protein organizes itself into the palm domain, composed of residues 239 to 315 arranged in six beta sheets, which are responsible for housing the His272 and Asp286 residues, components of the catalytic triad [76,78,79].

**Figure 8:**
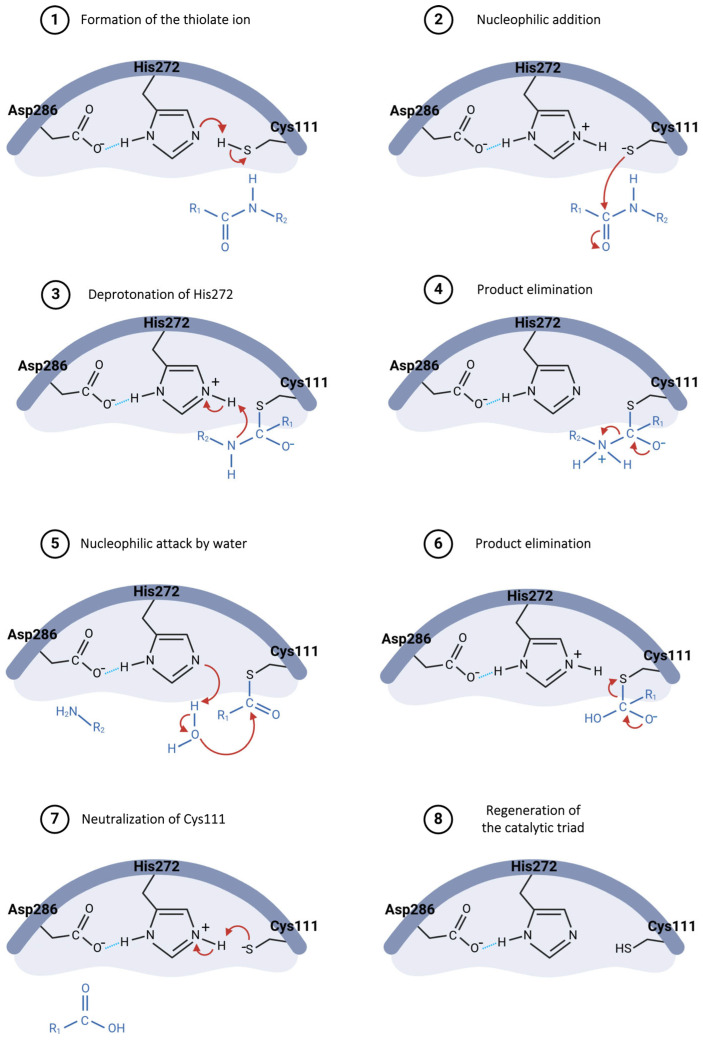
Proposed catalytic mechanism for SARS-CoV-2 PL^pro^ enzyme.

The hydrolysis mechanism catalyzed by the Pl^pro^ starts with Asp286 forming a hydrogen bond with the His272 residue, properly orienting this residue in the catalytic triad and enhancing its ability to abstract a proton from Cys111 (Figure 8, step 1) [73,80]. The resulting thiolate ion then attacks the carbonyl carbon of the peptide bond in the substrate (Figure 8, step 2), forming a tetrahedral intermediate, which is stabilized by the oxyanion hole within the active site [73,81]. His272, now acting as an acid, donates a proton to the amine group of the substrate (Figure 8, step 3), resulting in the release of the amino-terminal fragment (Figure 8, step 4) [82]. Following this, a water molecule is deprotonated by His272, generating a hydroxide ion that attacks the thioester bond between cysteine and the remaining substrate fragment (Figure 8, step 5), leading to another tetrahedral intermediate [80,81]. This intermediate then collapses, releasing the carboxy-terminal fragment of the substrate (Figure 8, step 6) and restoring the free cysteine thiol, ready to engage in the next catalytic cycle (Figure 8, steps 7 and 8) [82].

The cleavage process of non-structural proteins 1 and 2 occurs in an amino acid sequence recognized by the PL^pro^, which consists of the residues LXGG (nsp1 = LNGG↓AYTR and nsp2 = LKGG↓APTK), followed by self-cleavage at the interface corresponding to non-structural protein 4 (LKGG↓KIVN), leading to the modulation of the host immune response [73,75,77].

Two main pockets, S1 and S2, which embrace the substrate during the catalytic process, constitute the catalytic cavity of the PL^pro^ [72]. The high variability in the hydrophobic residues of the S2 pocket in the SARS-CoV-2 PL^pro^, compared to other coronaviruses, plays a crucial role in the enzyme’s specificity for the ISG15 substrate, whereas the S1 region is responsible for the enzyme’s significant catalytic activity [69,74]. During interaction with the protease, the *N*-terminal domain of interferon-stimulated gene 15 binds to the S2 pocket of the enzyme, while the *C*-terminal domain interacts with the S1 pocket in geometric orientations distinct from those observed for ubiquitin, aiding in understanding the enzyme’s substrate specificity [47,83,84]. At the present moment, there are no drugs available on the market targeting the SARS-CoV-2 PL^pro^ enzyme [80].

## 3. Development of Covalent Inhibitors Targeting SARS-CoV-2 Cysteine Proteases 

A literature review conducted by Barchielli, Capperucci, and Tanini (2024) on patents for protease M^pro^ and PL^pro^ inhibitors deposited between 2018 and 2024 revealed that the elucidation of enzyme structures through methods such as X-ray diffraction played a crucial role in the development of new molecules with potential therapeutic activity [37]. Most of these molecules contained warhead groups that facilitated the formation of a covalent bond with the protease, such as Michael acceptors, nitriles, thioketones, α-ketoamides, *trans*-α,β-unsaturated alkyl/benzyl esters, chloromethyl ketones, hydroxymethyl ketones, disulfides, and dithiocarbamates [37,85,86].

In recent decades, there has been progress in the field of medicinal chemistry, aiming at developing new covalent drugs for the treatment of cancer, viral infections, metabolic disorders, and gastrointestinal diseases [87,88]. Covalent bonds enhance the stability in the formation of the drug–receptor complex compared to non-covalent interactions, resulting in significant advantages such as prolonged drug action, the administration of lower doses of drugs, and increased treatment efficacy [89]. Moreover, covalent drugs are less susceptible to resistance caused by mutations, as the formed bond typically involves the nucleophilic residue essential for enzymatic catalysis [90,91].

The covalent inhibition mechanism occurs through two main steps: initially, the compound binds to the catalytic cavity of the enzyme, stabilized by non-covalent interactions [92,93]. These interactions facilitate the approach of the electrophilic moiety of the compound to the nucleophilic amino acid residue responsible for catalysis [87]. Subsequently, the previously established enzyme–inhibitor complex facilitates the formation of the covalent bond, which can be reversible or irreversible depending on the kinetic and structural characteristics of the compound (Equation (1)) [91,94].

(1) $$E+I\begin{array}{c} ⇌\\ K_{i} \end{array}\;E●I\;\begin{array}{c} k_{2}\\ ⇌\\ k_{-2} \end{array}E-I$$

where *E* denotes the enzyme, *I* is its inhibitor, *K*_i_ is the inhibition constant, *E.I* is the reversible enzyme–inhibitor complex, and *E-I* is the covalent enzyme–inhibitor complex. If the dissociation constant of the enzyme–inhibitor complex (*k*_-2_) is equal to zero—that is, smaller than the value of the formation constant (*k*_2_)—the correlation k* = *k*_-2_/*k*_2_ tends to infinity; thus, the reaction proceeds irreversibly [89,95]. However, if both values of *k*_2_ and *k*_-2_ are finite, the covalent bond formed can be reversible [87,96,97].

Despite the diversity of compounds developed as potential inhibitors of the M^pro^ enzyme, either through the addition of electrophilic moieties (warheads) or by the modulation of the allosteric sites of the protein, so far, only one orally administered antiviral has been approved by the Food and Drug Administration (FDA) for the treatment of COVID-19 by targeting the M^pro^ enzyme [62,98]. Paxlovid^®^, a medication developed by Pfizer, consists of a combination of two drugs: nirmatrelvir, a peptide mimetic that acts by covalently inhibiting the M^pro^ enzyme, and ritonavir, a CYP450 inhibitor that enhances the plasma concentration of nirmatrelvir [99,100].

### 3.1. Covalent Inhibitors Targeting SARS-CoV-2 M^pro^

**Figure 9:**
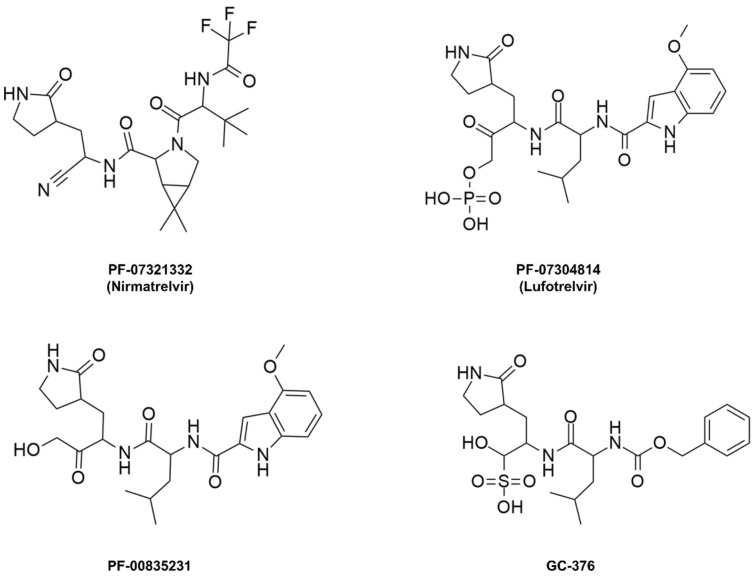
Covalent inhibitors of the M^pro^ enzyme derived from peptides.

**Nirmatrelvir**, initially named **PF-07321332**, is a peptide mimetic responsible for reversibly and covalently inhibiting the SARS-CoV-2 M^pro^ enzyme through a bond formed between the Cys145 residue of the protease and the nitrile moiety present in the compound’s structure [101,102,103]. This drug was designed based on peptide derivatives **lufotrelvir** (**PF-07304814**), a prodrug of **PF-00835231**, and **GC-376**, considered first-generation protease inhibitors, developed by Pfizer and by Anivive Lifesciences, respectively (Figure 9) [99,100,104].

**Figure 10:**
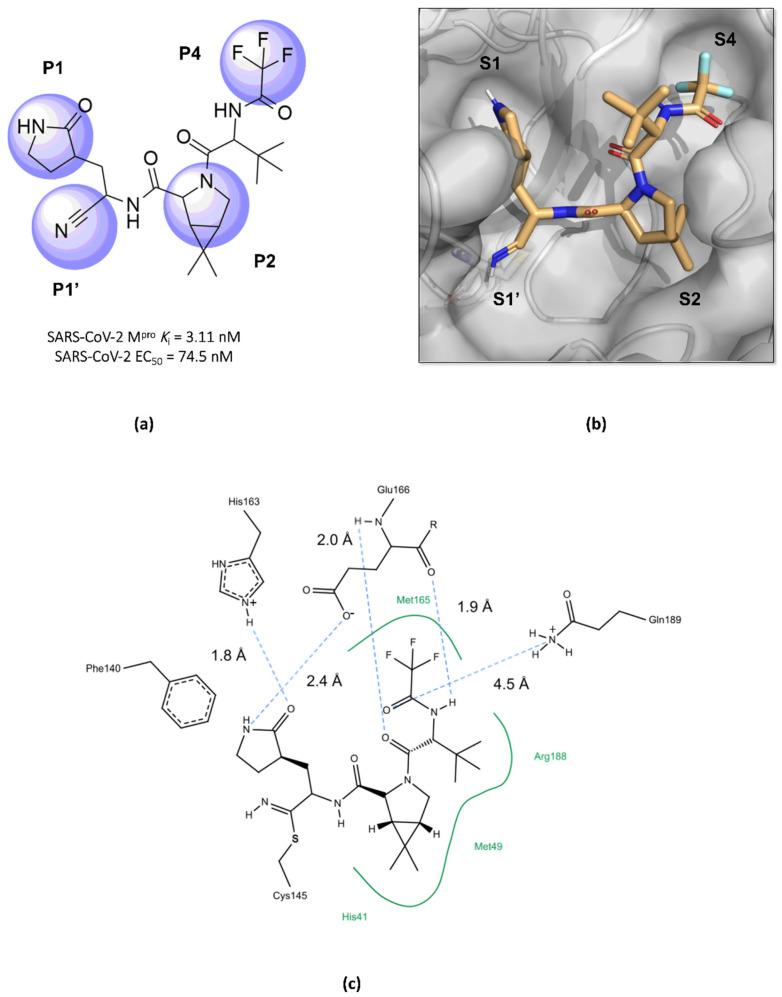
Main interactions of nirmatrelvir with SARS-CoV-2 M^pro^. (**a**) The 2D structure of nirmatrelvir, highlighting the substituents at positions responsible for interaction with the enzyme (in lilac). (**b**) The crystal structure of the M^pro^ complexed with nirmatrelvir (PDB ID 8DZ2), demonstrating the drug’s fitting into pockets located in the catalytic cavity of the protease. (**c**) Nirmatrelvir forms hydrogen bonds with the His163 residue (1.8 Å) and the Glu166 residue (2.4 Å), which help to stabilize the pyrrolidone ring in the S1 pocket, with assistance from the Phe140 residue. Additionally, the main chain of the Glu166 residue interacts with the carbonyl groups of the tert-butyl (2.4 Å) and trifluoroacetamide (1.9 Å) groups through hydrogen bonds, stabilizing the structure in the S4 pocket. A salt bridge is also formed between the side chain of Gln189 and the carbonyl group of the trifluoroacetamide group (4.5 Å). Furthermore, the residues His41, Met49, and Met165, and the main chain of Arg188 contribute to the stabilization of nirmatrelvir in the S2 and S4 pockets through hydrophobic interactions.

Structurally, nirmatrelvir features a pyrrolidone ring at the P1 position, mimicking the side chain of a crucial glutamine residue for interaction in the S1 pocket, followed by a warhead group composed of a nitrile. Moreover, there is a 6,6-dimethyl-3-azabicyclo[3.1.0]hexane ring at the P2 position, which fits into the S2 pocket, and it concludes with a trifluoroacetamide group accommodated in the S4 pocket (Figure 10) [96,99,101,105]. The inhibition constant (*K*_i_) value, which describes the binding affinity between nirmatrelvir and the enzyme, was determined as being 3.11 nM, while its EC_50_ value against SARS-CoV-2 was 74.5 nM [97,100]. However, due to its rapid metabolism by the enzyme CYP3A4, nirmatrelvir requires co-administration with ritonavir, a potent inhibitor of the cytochrome P450 enzyme complex, resulting in the significantly improved bioavailability of nirmatrelvir and the prolongation of its half-life [102,103,106].

**Figure 11:**
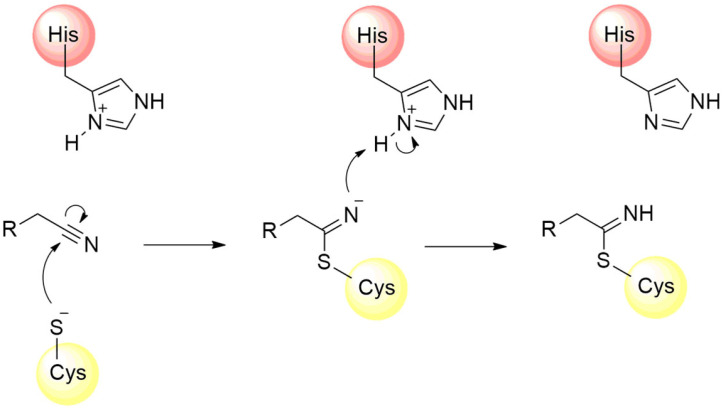
Mechanism of cysteine protease inhibition by nitrile-based inhibitors.

The design of several other covalent inhibitors of the SARS-CoV-2 M^pro^ containing diverse electrophilic groups has been achieved, significantly contributing to structure–activity relationship studies of the compounds [107,108,109]. In this context, after the clinical success of nirmatrelvir, the reactivity of the nitrile group towards the inhibition of the M^pro^ was also investigated by other authors. The nitrile group is found in various active compounds, contributing to the enhancement of their pharmacodynamic and pharmacokinetic parameters [110]. The presence of the nitrogen atom, associated with the dipolar moment induced by the triple bond, imparts an electrophilic characteristic to the carbon atom, rendering it susceptible to attack by nucleophilic residues, predominantly cysteines and serines [100,111]. Furthermore, the nitrogen atom of the nitrile can act as a hydrogen bond acceptor, thereby assisting in the stabilization of the compound within the enzyme’s catalytic cavity [110,112,113]. The mechanism of M^pro^ enzyme inactivation by the nitrile moiety is shown in Figure 11.

**Figure 12:**
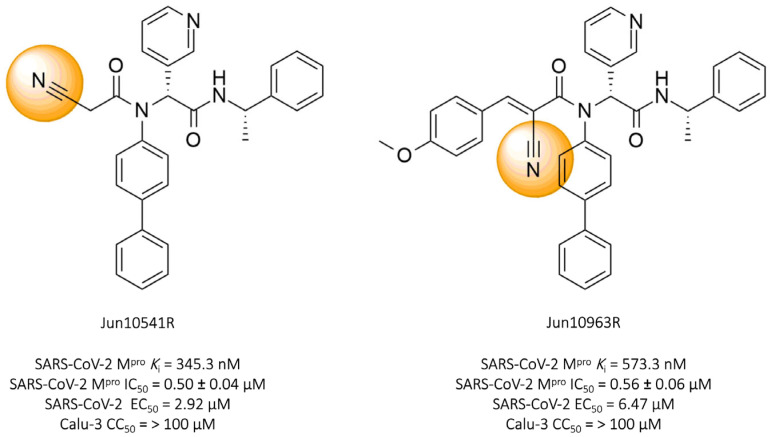
Compounds **Jun10541R** and **Jun10963R**: promising covalent inhibitors of SARS-CoV-2 M^pro^.

Tan and colleagues (2023) identified two promising nitrile-based compounds, **Jun10541R** and **Jun10963R**. The results showed that both compounds, **Jun10541R** (IC_50_ = 0.50 ± 0.04 μM, *K*_i_ = 345.3 nM) and **Jun10963R** (IC_50_ = 0.56 ± 0.06 μM, *K*_i_ = 573.3 nM), exhibited a significant ability to inhibit the SARS-CoV-2 protease, along with EC_50_ values against the virus of 2.92 μM and 6.47 μM, respectively, and cellular cytotoxicity (CC_50_) greater than 100 μM in Calu-3 cells (Figure 12) [114].

**Figure 13:**
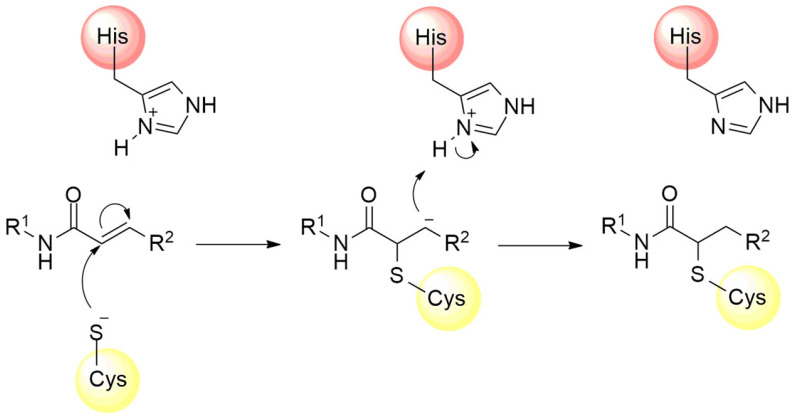
Mechanism of cysteine protease inhibition by acrylamide Michael acceptor.

In addition to the nitrile group, other electrophilic moieties can be used as warhead structures capable of binding cysteine residues. For example, compounds with electrophilic regions constituted by a Michael acceptor, such as α,β-unsaturated carbonyls, vinyl sulfones, and acrylamides, represent a promising approach in the development of new irreversible inhibitors against cysteine proteases [88,91]. These electrophilic groups feature an unsaturation at the β position of the carbon chain, rendering this region highly susceptible to nucleophilic attack by the cysteine residue, followed by subsequent proton capture at the α carbon [89,115,116] (Figure 13).

**Figure 14:**
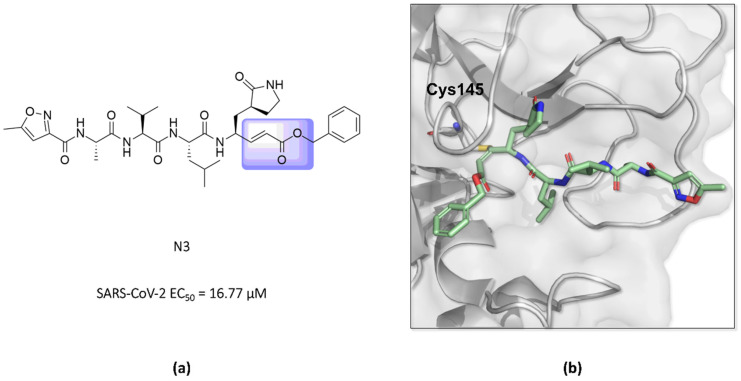
(**a**) Structure of the compound **N3** binding the SARS-CoV-2 M^pro^ via a Michael acceptor (in lilac). (**b**) The crystallographic structure shows that the peptide-like **N3** inhibits the SARS-CoV-2 M^pro^ enzyme via covalent binding with Cys145 (PDB ID 6LU7).

In this way, an inhibitor bearing a Michael acceptor as an electrophilic group has been described for the SARS-CoV-2 M^pro^ enzyme [117]. Previous studies have demonstrated the ability of the **N3** peptide to inhibit both the SARS-CoV-1 and MERS-CoV M^pro^ [118,119,120]. The antiviral activity assays of **N3** against SARS-CoV-2-infected Vero cells also confirmed its potent ability to inhibit the infection, with an EC_50_ value of 16.77 µM, suggesting that this compound can inhibit the M^pro^ enzyme of this virus, as seen for other β-coronaviruses [52,118]. Furthermore, the elucidation of the crystallographic structure of the formed complex confirmed the formation of a covalent bond between the sulfur atom of Cys145 in the SARS-CoV-2 M^pro^ and the β-vinyl carbon of the inhibitor (Figure 14) [43,52].

**Figure 15:**
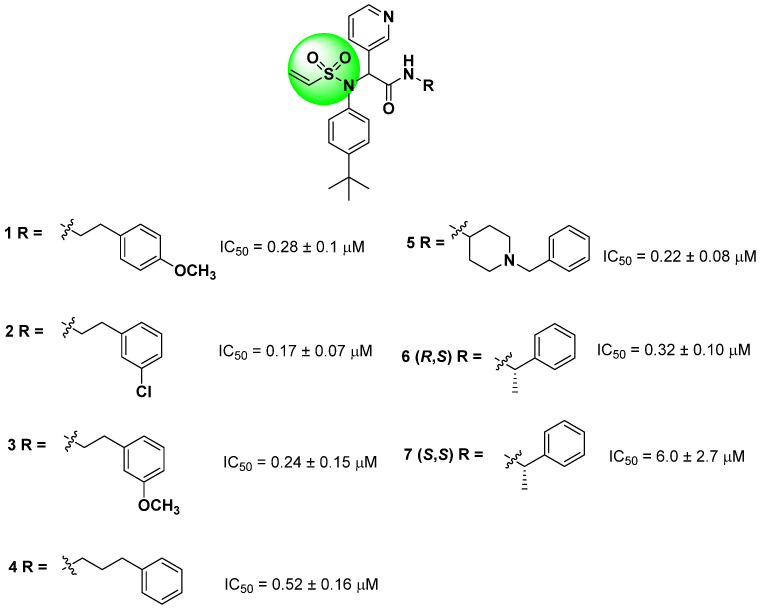
Structures of vinyl sulfonamide (in green) derivatives as covalent inhibitors of the SARS-CoV-2 M^pro^ and their corresponding IC_50_ values.

Starting from the non-covalent inhibitor co-crystallized with the SARS-CoV-2 M^pro^ enzyme (PDB ID 6W63), Stille and colleagues (2022) developed a series of covalent inhibitors containing a vinyl sulfonamide moiety (compounds **1–7**) as the electrophilic group [97,107,109]. The synthesized compounds demonstrated potent protease inhibition (>95%) when evaluated at 50 µM, with IC_50_ values lower than 6.0 ± 2.7 µM, as shown in Figure 15 [109].

**Figure 16:**
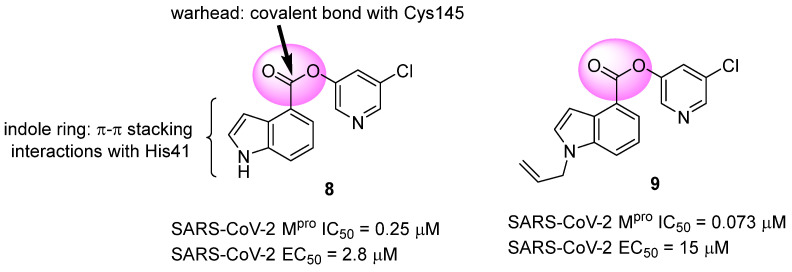
Indole ester derivatives as covalent inhibitors of the SARS-CoV-2 M^pro^, with the ester group (warhead) highlighted in pink.

Another approach explored in the development of covalent inhibitors targeting SARS-CoV-2 proteases involves the use of esters as the electrophilic group. Once the nucleophilic attack by Cys145 on the carbonyl carbon takes place, the hydroxyl-pyridine group is released as a leaving group, resulting in the formation of an irreversible covalent bond [97]. Previous studies conducted by Ghosh and colleagues (2008) demonstrated the SARS-CoV-1 M^pro^ inhibitory activity of indole ester **8**, with IC_50_ values of 0.03 ± 0.01 μM and an EC_50_ value against the virus of 6.9 ± 0.9 μM. In this study, the authors found that the presence of the 5-chloro-3-hydroxypyridine group was essential for the antiviral activity of the compound [121]. In this context, compound **8** was also tested against the M^pro^ of SARS-CoV-2, showing an IC_50_ of 0.25 μM and an EC_50_ of 2.8 μM in assays using infected Vero cells. The indole ester **8** exhibited antiviral activity comparable to remdesivir (EC_50_ = 1.2 µM) in the same assay [122,123]. Conversely, an *N*-allyl derivative **9** exhibited more effective enzyme inhibition, with an IC_50_ of 0.073 μM, despite showing lower activity against the virus (EC_50_ = 15 μM) (Figure 16). Subsequently, the inhibition mechanism elucidated by X-ray crystallography confirmed the release of the 5-chloro-3-hydroxypyridine group as a leaving group after the enzyme attack [122].

**Figure 17:**
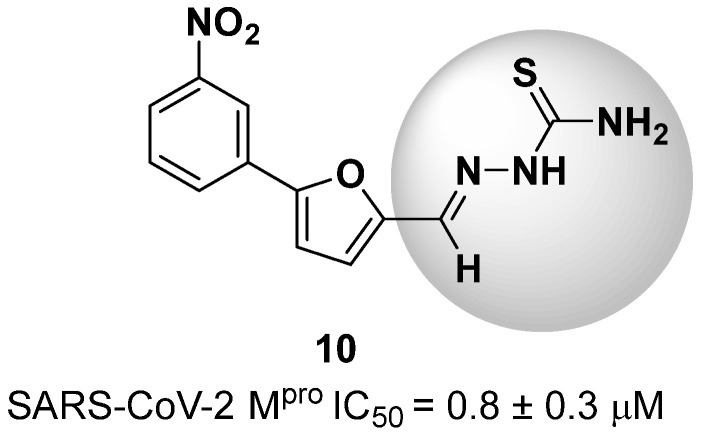
Arylfuran derivative **10** containing a thiosemicarbazone moiety (in gray) as a reversible covalent inhibitor of the SARS-CoV-2 M^pro^.

New warhead groups are being investigated for the design of drug candidates aimed at treating COVID-19. A study conducted by Maltarollo and colleagues (2023) using virtual screening identified 62 hits, four of which contained the thiosemicarbazone moiety. These compounds demonstrated the reversible covalent inhibition of protease activity amounting to 100% when evaluated at 10 μM, with the arylfuran derivative **10** showing an IC_50_ of 0.8 ± 0.3 μM against the M^pro^ enzyme of SARS-CoV-2. These results suggest some selectivity compared to the SARS-CoV-1 protease, for which the IC_50_ value was 2.2 ± 1 μM, indicating potential differentiation between the two related viruses (Figure 17) [124].

**Figure 18:**
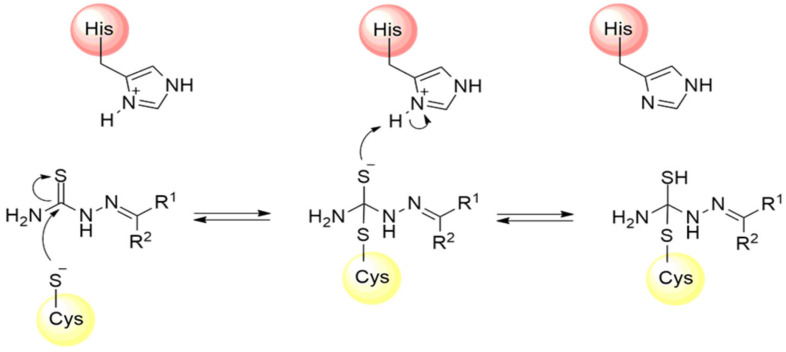
Mechanism of cysteine protease inhibition by thiosemicarbazones.

Thiosemicarbazones are a class of compounds widely studied as inhibitors of cysteine proteases [125,126,127]. The mechanism of inactivation of these compounds involves the formation of a transient covalent bond with the catalytic residue of cysteine through the electrophilic carbon present in the thiosemicarbazone group (Figure 18) [94].

### 3.2. Covalent Inhibitors Targeting SARS-CoV-2 PL^PRO^

**Figure 19:**
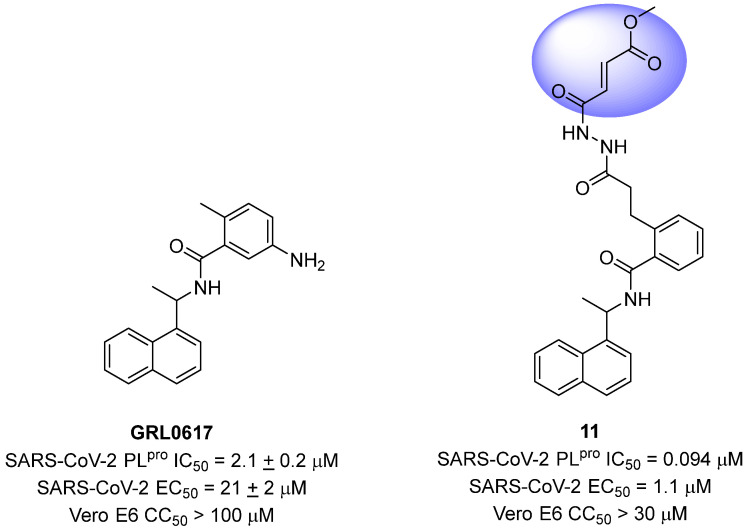
Michael acceptor derived from **GRL0617** as a SARS-CoV-2 PL^pro^ covalent inhibitor, highlighting the reactive electrophilic moiety (fumarate ester).

Sanders and colleagues (2023) have developed inhibitors against the SARS-CoV-2 PL^pro^ based on Michael acceptors. An analysis of the non-covalent inhibitor **GRL0617** co-crystallized with the SARS-CoV-2 PL^pro^ revealed that the phenylmethyl group present in the ligand structure was positioned at a distance of 7 Å from the sulfur atom of Cys111 in the catalytic site of the protease. Therefore, to establish a covalent bond with the PL^pro^ and preserve the interactions already established with the enzyme, a linker, *N,N’*-acetylacetohydrazine, resistant to protease hydrolysis, was added to the ligand structure and fused with the electrophilic methyl fumarate ester group (compound **11**) [72,128] (Figure 19). This modification resulted in an enzyme inactivation rate (*k*_inact_) relative to the inhibition constant (*K*_i_) of 9600 M^−1^ s^−1^, with an IC_50_ value of 0.094 μM [85]. Furthermore, infection assays with SARS-CoV-2 in Vero E6 cells demonstrated notable antiviral activity promoted by the compound, with an EC_50_ value of 1.1 μM, and no evidence of cellular cytotoxicity (CC_50_ > 30 μM) associated with the inhibition of human deubiquitinases (DUBs) [128].

**Figure 20:**
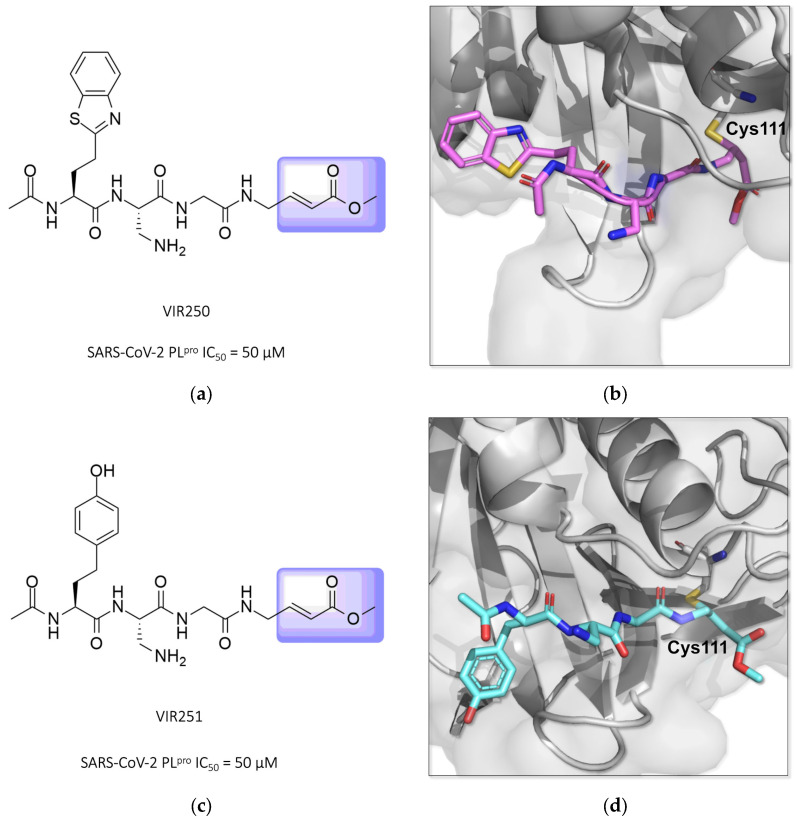
Structures of the compounds **VIR250** and **VIR251** binding the SARS-CoV-2 PL^pro^ enzyme, highlighted in lilac for the α,β-unsaturated ester (Michael acceptor). The crystallographic structure shows that compounds **VIR250** (PDB ID 6WUU) (**a**,**b**) and **VIR251** (PDB ID 6WX4) (**c**,**d**) inhibit the SARS-CoV-2 PL^pro^ enzyme via covalent binding with Cys111.

Two other potent covalent Michael acceptor-based inhibitors described for the SARS-CoV-2 PL^pro^ enzyme were reported in studies conducted by Rut and colleagues (2020) [129,130]. The compounds **VIR250** and **VIR251**, both peptide mimetics, were able to inhibit the proteases of both SARS-CoV-1 and SARS-CoV-2 and showed no activity against human deubiquitinase UCH-L3 (Figure 20) [84,130,131,132].

## 4. Development of Non-Covalent Inhibitors Targeting SARS-CoV-2 Cysteine Proteases

The majority of the cysteine protease inhibitors are covalent, but, due to the necessary reactivity to form a covalent bond with cysteine residues, they may be non-specific and react with other nucleophiles rather than the intended enzyme, resulting in the possibility of adverse effects [133]. This principle highlights the importance of the search for and development of non-covalent inhibitors of the M^pro^ and PL^pro^.

In this context, it is possible to observe that most published inhibitors are covalent and only a few are non-covalent. A search on PubMed of the terms “(covid19) AND (covalent inhibitor) AND (main protease) OR (3CLpro) OR (papain-like protease)” yields 347 results, with 38 in 2020, 76 in 2021, 94 in 2022, 90 in 2023, and 49 in 2024. On the other hand, when the term covalent inhibitor is substituted for non-covalent “(covid19) AND (non-covalent inhibitor) AND (main protease) OR (3CLpro) OR (papain-like protease)”, the total results are reduced by 66% compared to the first search, leading to 118 results, with 13 in 2020, 31 in 2021, 33 in 2022, 25 in 2023, and 16 in 2024 (Table 1).

**Figure 21:**
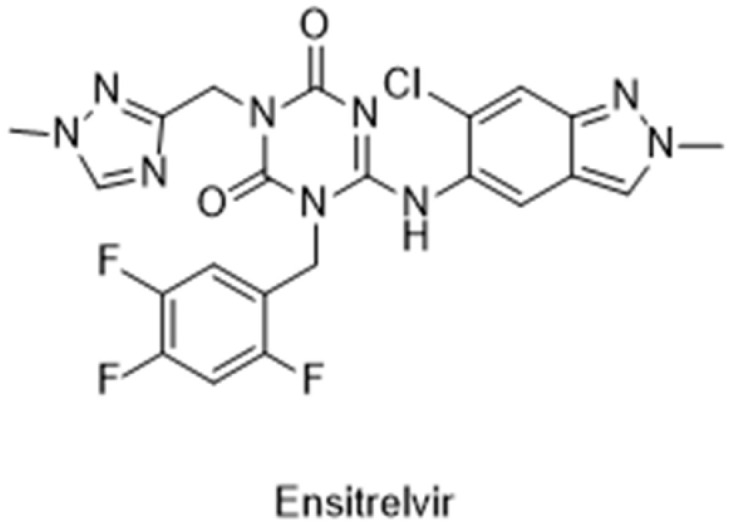
Structure of ensitrelvir, the first non-covalent M^pro^ inhibitor approved.

The Japanese Ministry of Health, Labour and Welfare (MHLW) approved the first non-covalent M^pro^ inhibitor, ensitrelvir (trade name Xocova^®^) (Figure 21), under emergency regulatory conditions, only in November 2022 [134,135]. Furthermore, in May 2023, the company submitted an application for standard approval, which was conceded on 5 March 2024. On the other hand, the use of covalent inhibitors started in December 2021, almost a year before, with the FDA emergency use authorization of Paxlovid^®^, a combination of nirmatrelvir, the covalent inhibitor of the M^pro^, and ritonavir, a potent CYP3A inhibitor [101,102,103].

### 4.1. Non-Covalent Inhibitors Targeting SARS-CoV-2 M^pro^

**Figure 22:**
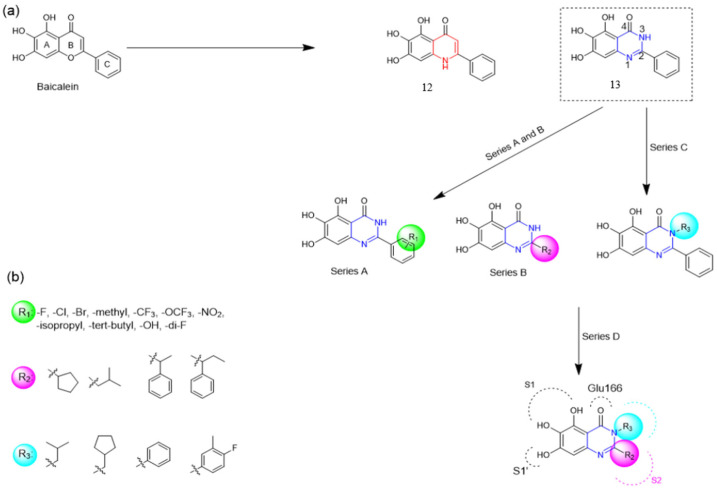
(**a**) Detailed optimization strategy for the design of non-covalent SARS-CoV-2 M^pro^ inhibitors from baicalein and indications of the series of new compounds. (**b**) Substituents used in positions R1, R2, and R3.

Henceforth, the discussion in this review will focus on the most recent advances in the development of non-covalent inhibitors of the M^pro^. Zhang and colleagues (2023) started from baicalein, a flavonoid described as a non-covalent M^pro^ inhibitor, and proceeded with structural modifications of this lead structure (Figure 22) in order to achieve new and more potent M^pro^ non-covalent inhibitors [136,137]. These authors established a structure–activity relationship (SAR) study for the synthesized and tested compounds, besides the evaluation of their metabolism and pharmacokinetic (DMPK) properties [136].

Based on the baicalein structure, novel scaffolds were predicted using the scaffold hopping technique, and the chromen-4-one core was replaced with a quinazolin-4-one (Figure 22). Some requirements were considered to propose the pharmacophore features: (i) the presence of a hydrogen bond acceptor that can interact with the NH at the side chain of His163 in the S1 sub-pocket; (ii) the presence of a hydrogen bond acceptor that can interact with NH at the main chain of Glu166; and (iii) the presence of a hydrophobic group that can interact with the S2 sub-pocket. The three phenolic hydroxyls and the carbonyl group of baicalein were shown to be essential to the activity, being maintained in the quinazolinone analogues, which formed a critical hydrogen bond. The compound **12**, which only changed the O atom in the B ring with an N atom, had no inhibitory activity against the M^pro^. In contrast, the quinazoline derivative **13**, which had another N atom, had similar inhibitory activity to baicalein (Table 2). Because of this, **13** was chosen for further modifications and optimization. The chosen positions for the variations in the substitutions were the C2, with R2 substituents, and N3, with R3 substituents [136].

Initially, two series of compounds were synthesized by varying the substituent at C2: (i) series A—mono- or disubstituted benzene rings; (ii) series B—aromatic/heteroaromatic rings, alkyl groups, or a side chain with both alkyl and phenyl groups (Figure 22). In series A, the substituent R1 was an electronegative group (F, Cl, Br, CF_3_, OCF_3_, NO_2_, OH, and di-F) or an alkyl group (methyl, isopropyl, and *tert*-butyl) positioned at different positions in the aromatic ring. The most impactful parameters for the activity against the M^pro^ were the polarity and size of the groups. Less bulky groups with moderate to low electronegativity have been shown to positively influence the activity, with isopropyl, *tert*-butyl, hydroxyl, and nitro groups associated with an accentuated decline in activity. The most potent compounds in this series of modifications were compounds **A4** and **A10** (Table 2), with IC_50_ values of 0.435 ± 0.04 μM and 0.365 ± 0.033 μM, respectively, with similarly sized and low-electronegativity substituents (Cl and Me) at the *ortho* position. Thus, it was described that the position, steric size, polarity, and electronic properties of the groups were the factors that had major importance for the inhibitory activity [136].

The next parameter evaluated was the replacement of the phenyl ring (C ring) with other groups (series B) (Figure 22). When performed directly with naphthyl and heteroaromatic rings, it led to a decrease in the potency of the molecules. Cycloalkyl rings were also tested, having various impacts on the potency of the compounds, with the cyclopentyl ring being the better group (compound **B7**). In addition, non-cyclic alkyl groups were also evaluated, and the better-performing one was the *sec*-butyl group (compound **B11**). However, compounds with a phenyl ring separated from the quinazolinone core by a C atom, namely compounds **B15** and **B16**, were shown to be most potent, with IC_50_ values of 0.174 ± 0.038 μM and 0.210 ± 0.028 μM, respectively [136].

In the other series studied (series C), the structural changes were now focused on the substituents linked to the N-3 (R3 substituents) (Figure 22). For standardization reasons, the phenyl group was maintained at the C-2 position. In general, the introduction of substituents at N-3 resulted in compounds with greater potency than **13**, with the most potent being compound **C7** (Table 3), which possessed a phenyl ring attached to nitrogen (IC_50_ = 0.085 ± 0.006 μM). Other compounds with comparable inhibitory activity were **C5** and **C1**, both being slightly less potent than **C7**, bearing, in the N-3 position, respectively, cyclopentyl and *sec*-butyl groups, which are mildly bulky and hydrophobic groups [137].

To conclude the SAR study, the last series was the D series (Figure 22), in which the best substituents of both positions, C-2 and N-3, were included. Initially, the best combination by logic, with the best substituents in each position, namely C-2 and N-3, decreased the potency of the inhibition four-fold (compound **D1**). The hypothesis described to explain this was that the groups used—for C-2, 2-methyl-benzyl (compound **B15**), and for N-3, phenyl/*sec*-butyl (compounds **C1/C7**)—competed with each other for space in the enzyme pocket and neither of them interacted appropriately with the M^pro^. In this series, the most potent compound was **D8** (IC_50_ = 0.100 ± 0.012 μM), followed by **D6** (0.107 ± 0.023 μM), both having an aromatic ring in N-3, but with compound **D8** having a *sec*-butyl group in C-2 and compound **D6** a *tert*-butyl group in the same position (Table 3) [136].

Additionally, pharmacokinetic studies were performed with the three most promising compounds, **C7**, **D6**, and **D8**, in comparison with baicalein. These compounds exhibited better membrane permeability in canine kidney cells (Madin–Darby canine kidney—MDCK) and higher kinetic solubility in phosphate buffer solution (PBS, pH = 7) compared to baicalein. All tested compounds showed low plasma protein binding (PPB) in human plasma (**C7**: 0.70%, **D6**: 2.61%, **D8**: 3.17%, and baicalein: 0.93%). Moreover, the clearance rate (CLint) in human liver microsomes (HLMs) was measured, with the baicalein CLint = 333.05 μL/min/mg protein and a half-time (t_1/2_) of 4.16 min. The compounds **C7** (CLint = 108.68 μL/min/mg protein and t_1/2_ = 12.75 min) and **D6** (68.34 μL/min/mg protein and t1/2 = 20.28 min) showed lower clearance and a higher half-life value than baicalein (Table 4). The combination of these results demonstrates that these compounds showed good druggability [136].

Finally, cytotoxicity and antiviral assays were carried out in Vero E6 cells (Table 5). None of the tested compounds (**C7**, **D6**, **D8**) showed a significant impact on the cell viability (CC_50_ > 50 μM), including baicalein (positive control). In the inhibitory evaluation using the viral replication assay, Vero E6 cells infected with SARS-CoV-2 were incubated for 1 h with the compounds, followed by 1 h of infection. The cells were then incubated for 48 h and the quantification of the RNA copy number of the virus was performed by RT-qPCR. The EC_50_ was, in this case, the concentration of the compound equivalent to a 50% number of RNA copies compared to no inhibition. Among all tested compounds, **C7** (EC_50_ = 1.10 ± 0.12 μM), **D6** (EC_50_ = 2.87 ± 1.43 μM), and **D8** (EC_50_ = 2.11 ± 1.12 μM) showed better antiviral activity than baicalein (EC_50_ = 5.15 ± 1.64 μM) (Table 5). These results indicate quinazolin-4-one as a promising new scaffold for the development of new non-covalent inhibitors of the M^pro^. In response to this scenario, the next steps would be structural modifications aiming for better DMPK performance, which could, then, advance to in vivo studies and clinical trials [136].

**Figure 23:**
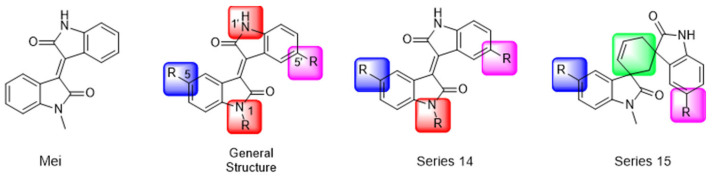
Structure of mesoindigo (Mei), general structure of the scaffold used in the structural optimization and scaffolds of series 14 and 15.

Another recent discovery in the development of non-covalent M^pro^ inhibitors was described in 2024 by Gao and colleagues [138]. Enzymatic assays, the inhibition of SARS-CoV-2 replication, and gastrointestinal and metabolic stability studies were conducted for mesoindigo (Mei) derivatives (Figure 23), and promising results were achieved. Mei is a mono *N*-methyl derivative of indirubin extracted from *Indigo naturalis*, displaying various types of biological activity, such as anti-inflammatory [139] and antitumor [140] activity. However, studies about its antiviral activity had not been conducted before. This scenario motivated further investigations of Mei as a lead compound for the design of non-covalent M^pro^ inhibitors.

Initially, a screen with a library of traditional Chinese medicines (TCMs) composed of natural products was performed to detect novel SARS-CoV-2 M^pro^ inhibitors. From this initial screening, Mei was identified as a hit, able to inhibit the activity of the M^pro^ with an IC_50_ value of 15.65 μM [138].

The main modifications in the Mei structure that were explored were *N*-alkylations at positions *N-*1 or *N-*1′ (highlighted in red in Figure 23) and the variation of the substituents on the phenyl ring at positions C-5 or C-5′ (highlighted in blue/pink in Figure 23). These two groups of compounds belong to series 14, while the molecules that have an extra-condensed ring (highlighted in green in Figure 23), in addition to the other possible substituents, belong to series 15 [138].

The substituents used in the C5/C5′ of the aromatic ring were -NO_2_, Cl, Br, F, MeO, and HO-, among which the last three improved the inhibitory potency of the compounds, and the first three reduced their activity, when compared to Mei. On the other hand, *N*-alkylation with different groups resulted in a variety of effects on the inhibitory activity. Compound **S14-1**, containing no alkyl substituent at *N*-1, showed better activity than Mei (Table 6), resulting in a structure similar to that of glutamine (Gln), which is recognized by the S1 site of the M^pro^ [138].

The enzymatic inhibition assays were performed using ebselen as a positive control. The compounds were tested at a 10 µM concentration. Among the 31 compounds tested, 18 exhibited M^pro^ inhibitory activity higher than 70%, of which six inhibited over 90%, with an emphasis on compound **S14-3**, capable of inhibiting 97.8% of the enzyme activity. On the other hand, the compounds from series 15 did not show inhibition over 12%, indicating that the condensed rings, with the disruption of the conjugated system, had no effect in inhibiting the protease activity [138].

Next, the concentration-dependent inhibition was tested for the six most active compounds, **S14-1**, **S14-2**, **S14-3**, **S14-4**, **S14-5**, and **S14-6**, and the values of the IC_50_ were determined (Table 6) as, respectively, 1.20 µM, 1.15 µM, 1.21 µM, 1.61 µM, 1.09 µM, and 1.35 µM. In addition, enzymatic inhibition kinetic analyses were conducted for the compounds **S14-5** and **S14-6**, with 10, 30, and 50 min of pre-incubation of the compounds with the protein. The results indicated that these derivatives had a reversible and not time-dependent type of interaction with the M^pro^, which makes them non-covalent inhibitors [138].

The cytotoxicity of the compounds from series 14 was evaluated at 10 µM in human embryonic kidney 293 cells, and none of them showed significant effects on cell viability. After these results, the ability of the compounds to inhibit SARS-CoV-2 replication was evaluated in Calu-3 and HeLa-hACE2 cells stably expressing angiotensin-converting enzyme 2 (ACE2). Using a Western blot and qRT-PCR, the concentrations of gene expression of the spike protein (Protein S) and nucleocapsid were determined, being the metric used to calculate the viral replication inhibition. The EC_50_ values of the compounds were the concentrations that decreased these proteins’ relative levels by 50%. Compounds **S14-3** and **S14-7** were tested against Calu-3 cells and showed significant activity. The antiviral activity in HeLa-hACE2 cells was assayed with the compounds **S14-2**, **S14-5**, and **S14-6** using nirmatrelvir as a positive control, and the results are shown in Table 7 [138].

Finally, the authors evaluated the physicochemical properties and gastrointestinal stability for the compounds **S14-2**, **S14-5**, and **S14-6**, and the metabolic stability under exposure to mouse liver microsomes of **S14-6**. Firstly, the water solubility of these three compounds was determined, and the results were 17.31, 3.81, and 2.49 mg/mL, respectively. The LogP was also experimentally assessed, resulting in −0.70, −0.59, and −0.29. In addition to these results, their stability in simulated gastric and intestinal fluid was determined (Table 8) through HPLC analysis. **S14-6** was considered the most promising compound in this study, because it demonstrated high potency against both the enzyme and the virus and high stability in the intestinal and gastric fluids, so it was chosen to be further investigated upon exposure to mouse liver microsomes. **S14-6** was incubated with the cells, and samples were collected at time points 0, 30, 60, 90, and 120 min and quantified by HPLC, resulting in 84% of the unaltered compound after 2 h of exposure. Moreover, the hepatic microsomal metabolic half-life (t_1/2_) and clearance (Cl_int_) were theoretically calculated, resulting in 495 min and 1.4 μL min^−1^mg^−1^, respectively. These results attest to the druggability of these compounds [138].

### 4.2. Non-Covalent Inhibitors Targeting SARS-CoV-2 PL^pro^

**Figure 24:**
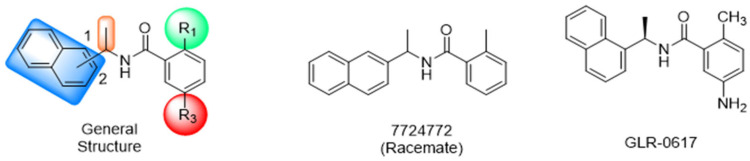
General structure of the scaffold used in structural optimization, based on the structure of 7724772 and the structure of GLR0617.

Historically, the development of non-covalent inhibitors of the PL^pro^ started in 2008, leading to the most influential compound in the initial stages of the development of non-covalent PL^pro^ inhibitors, compound **GLR0617** (Figure 24) [72,133]. Initially, 50,080 compounds were screened to find the most promising molecules. The evaluation of the inhibitory activity was performed using a technique developed by our own group [141]. Among the compounds tested, only 17 (0.014% of total) showed over 35% inhibition against the PL^pro^. Compound **7724772** (Figure 24), a racemic mixture of 2-methyl-*N*-[1-(2-naphthyl)ethyl]benzamide, inhibited the PL^pro^ with an IC_50_ of 20 ± 1.1 µM, being chosen for structural optimization. In order to investigate the influence of the stereogenic center, both enantiomers (*R*) and (*S*) were tested at a concentration of 100 µM. The (*S*) enantiomer inhibited only 14% of the enzyme activity, while the (*R*) inhibited over 90%, with an IC_50_ value of 8.7 ± 0.7 µM. Then, the (*R*) enantiomer was selected as a hit compound for further investigation [133].

The general structure shown in Figure 24 was divided into three points of variation: (i) the pattern of substitution in the naphthalene ring, (ii) the substituent in -*ortho* (R_1_) in the benzene ring, and (iii) the substituent in -*meta* (R_2_) in the benzene ring (compounds **15**–**19**). The first conclusion was that a methyl group in R_1_, instead of an H atom in this position, is better for the activity, based on the results of the previous screening. After this, because of its similar size to the methyl, a chlorine atom was considered in R_1_ (compound **15**) (Table 9), but it decreased the inhibitory activity by half. Still in this position, a bulker group (ethyl) was considered (compound **16**), but it completely abolished the activity against the PL^pro^. Next, the substitution pattern of the naphthyl ring was varied from 2-naphthyl to 1-naphthyl. This change, keeping the methyl group at R_1_, resulted in compound **17**, which had four times higher potency than **7724772**. Finally, the variation of the substituent in R_3_ (R_3_ = H, NHAc, NO_2_, and NH_2_) was evaluated and the compound **GLR0617**, containing an amino substituent, was shown to be the most active of the series against the PL^pro^, with an IC_50_ value of 0.6 ± 0.1, which is four times lower than that of compound **17**. This suggests that a new hydrogen bond or a new ionic bond can be established with the molecular target [133].

After these processes, the antiviral activity of the compounds was assayed in Vero E6 cells infected with SARS-CoV. Compounds **GLR0617**, **17**, and **18** had the best results, with an EC_50_ value from 10 to 15 µM and no cytotoxicity in uninfected cells up to a concentration of 50 µM (Table 9) [133].

These results, despite being described for SARS-CoV in 2008, 11 years before the emergence of COVID-19 and SARS-CoV-2, made a significant contribution to the development of new non-covalent inhibitors of the PL^pro^, reinforcing the idea that there is high genetic conservation among coronaviruses.

**Figure 25:**
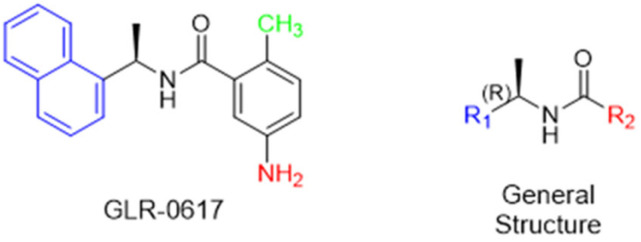
Structure of **GLR0617** and general structure of scaffold used in structural optimization.

Starting the review of the most recent PL^pro^ non-covalent inhibitors, Garland and colleagues (2023) carried out the large-scale virtual screening of the ZINC20 database, with the deep docking (DD) technique, in sub-pockets S3 and S4 of the catalytic site of this enzyme [142]. In order to validate the process, self-docking simulations with two non-covalent inhibitors of the PL^pro^ already described in the literature, **GLR0617** [72,133] and **XR8-89** [131], were performed. The in silico screening resulted in two hit selection strategies. The first was based on a six-point pharmacophore model. After ranking the compounds from this model, 200 compounds were selected for experimental validation. The second hit list was based on a four-point pharmacophore model. From this list, the compounds were docked to the PL^pro^ in ICM and Glide, and 60 compounds had high scores in both programs and were selected for experimental validation. From 260 compounds, 178 were screened against the PL^pro^ (Table 10). The compounds were variations of the **GLR0617** structure, following the pattern shown in Figure 25. The changes generated the VPC series (Table 10) [142].

Seventeen compounds were able to inhibit more than 30% of the PL^pro^ activity at 130 µM in the enzymatic assay. The compounds identified as the most active were **VPC-300141** (100% inhibition), **VPC-300195** (100% inhibition), **VPC-300016** (70% inhibition), and **VPC-300002** (65% inhibition). These compounds, alongside **GLR0617** and **XR8-89**, had their PL^pro^ IC_50_ values determined (Table 10) [135].

Next, these four compounds were further investigated in an in vitro assay with replication-competent SARS-CoV-2 (Wuhan Hu-1 clone) and Vero E6 cells constitutively expressing TMPRSS2. In this assay, nirmatrelvir, the active component of Paxlovid^®^ [64,101,102,103], was used as a control for complete inhibition, together with **XR8-89**. Vero cell lines generally express high levels of the p-glycoprotein efflux transporter, which can decrease the antiviral activity of some compounds [143]. In this scenario, the first condition of the assay had no addition of the efflux transporter inhibitor **CP-100356**, meaning that the efflux rate was unaltered, and the compounds were pre-incubated with the cells for 0, 1, and 3 h. In the second assay condition, the efflux transporter was inhibited and the compounds were not pre-incubated with the cells (0 h) [142].

At time 0 and 1 h, all molecules tested, except nirmatrelvir, showed an IC_50_ higher than 100 µM. However, after 3 h pre-incubation, **XR8-89** and **VPC-300195** showed an IC_50_ of 32.0 µM and 17.9 µM, respectively. Notwithstanding, when the transporter inhibitor was added, the assay was carried out without the pre-incubation of the molecules. In this case, all molecules showed IC_50_ values lower than 100 µM, with the three highest-ranked compounds being nirmatrelvir (IC_50_ = 3.9 µM), **VPC-300195** (IC_50_ = 15.0 µM), and **XR8-89** (IC_50_ = 40.3 µM) (Table 11) [142].

The results in both conditions (with and without the transporter inhibitor) showed that **VPC-300195** had higher potency in inhibiting the PL^pro^ when compared to the already described **XR8-89**, which was considered a potent non-covalent inhibitor. Consequently, the authors associated the higher activity of this molecule with the presence of the sulfonamide group in the -*para* position to the amide group, which interacts with the PL^pro^ S3 sub-pocket, potentially being a starting point for new drug design studies [142].

### 4.3. Non-Covalent Inhibitors Targeting Both SARS-CoV-2 M^pro^ and PL^pro^

**Figure 26:**
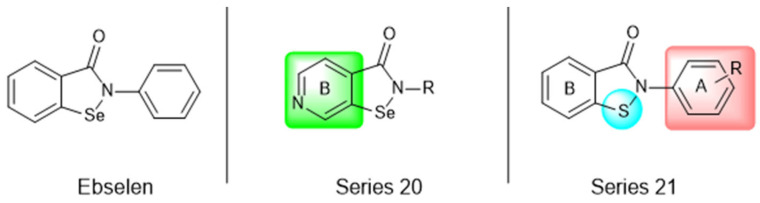
Structure of ebselen and general structures of series 20 and 21.

Most recently, Liu and colleagues (2024) studied a scaffold of benzo[d]isothiazol-3(2H)-one and identified a new, promising structure for the development of compounds capable of inhibiting both the PL^pro^ and M^pro^ [144]. Ebselen, a well-known M^pro^ covalent inhibitor [145], was used as a starting point for further modifications: (i) the introduction of various substituents in the A ring; (ii) the replacement of the B ring with pyridine, leading to series 20; and (iii) the replacement of the Se with S, leading to series 21, in order to obtain non-covalent inhibitors (Figure 26). In addition, it was discussed that the bond N-Se was unstable, which was another motivation for the study to change it for the N-S bond. This is caused by the cleavage of the N-Se bond during the nucleophilic attack by the cysteine residue at the enzyme’s active site, which is only required for covalent inhibitors. Thus, replacement with a group that did not covalently bind to the enzyme was necessary. Its resistance to the attack of cysteine can be explained by the shorter bond length (N-S < N-Se), indicating higher bond energy (E_N-S_ > E_N-Se_) [144].

After synthesizing 32 compounds, compounds **21a–c**, described as potent non-covalent inhibitors of the PL^pro^, presented the most promising results among the entire series after being screened against the PL^pro^ at the concentrations of 5000, 500, and 50 nM and measuring their percentage inhibition of the enzyme, using **GLR0617** and ebselen as positive controls (Table 12).

Due to the already known inhibitory activity of ebselen against the M^pro^ [52], the compounds **21a–c** were also tested against this protease in order to determine whether the replacement of the Se atom would result in a change in the mechanism of inhibition to a non-covalent one. The enzymatic inhibitory activity of the compounds was evaluated and compounds **21a** and **21c** had slightly better performance than ebselen, inhibiting 50% of the M^pro^ activity at lower concentrations than 500 nM, while the control inhibited only 43.1% at the same concentration (Table 13).

Subsequently, the cytotoxicity of the compounds **21a–c** and their ability to inhibit SARS-CoV-2 replication in Vero E6 cells was tested. Initially, the molecules were incubated with non-infected Vero E6 cells at the concentrations of 1.5, 3.125, 6.25, 12.5, 25, and 50 µM for 48 h. All of the compounds exhibited elevated cytotoxicity at the concentrations of 25 and 50 µM, limiting the cell viability at 40%. Despite this, these compounds were tested in infected Vero E6 cells. Compounds **21a** and **21c** successfully inhibited viral replication, with comparable potency to that of ebselen and higher than that of **GLR0617** (Table 14) [144].

The study also performed a liver homogenate metabolic stability assay with compound **21c**. The authors chose this assay because it has already been further investigated in molecular docking and dynamic simulations. Thus, compound **21c** was incubated with the liver homogenate mixture for 48 h to evaluate its stability under oxidative metabolism. In the defined time period, an aliquot was quenched and analyzed through HPLC with a UV detector at 254 nm and the remaining unaltered compound was measured. As shown in Table 15, in the first 12 h, there were still 70.6% ± 1.4 of the compound, while, at 24 h, 53.6% ± 2.1 was still available, indicating that **21c** has a half-life longer than 24 h in these conditions. Lastly, at 48 h, there was only 28.9% ± 2.9 of unaltered **21c**. These results pave the way for new in vivo studies of these and similar compounds [144].

## 5. Perspective

The most recent discovery for the treatment of COVID-19 is the peptide-like inhibitor **simnotrelvir** (**SSD8432** or **SIM0417**), an oral covalent inhibitor designed from boceprevir [146,147]. This drug was developed by the Shanghai Institute of Materia Medica and the Wuhan Institute of Virology and approved by the National Medical Products Administration in China. Simnotrelvir is available for oral administration in tablet form (trade name XIANNUOXINTM), which contains simnotrelvir 0.75 g (0.375 g × 2) and ritonavir 0.1 g, and is used to treat patients with mild to moderate COVID-19 [146,148].

**Figure 27:**
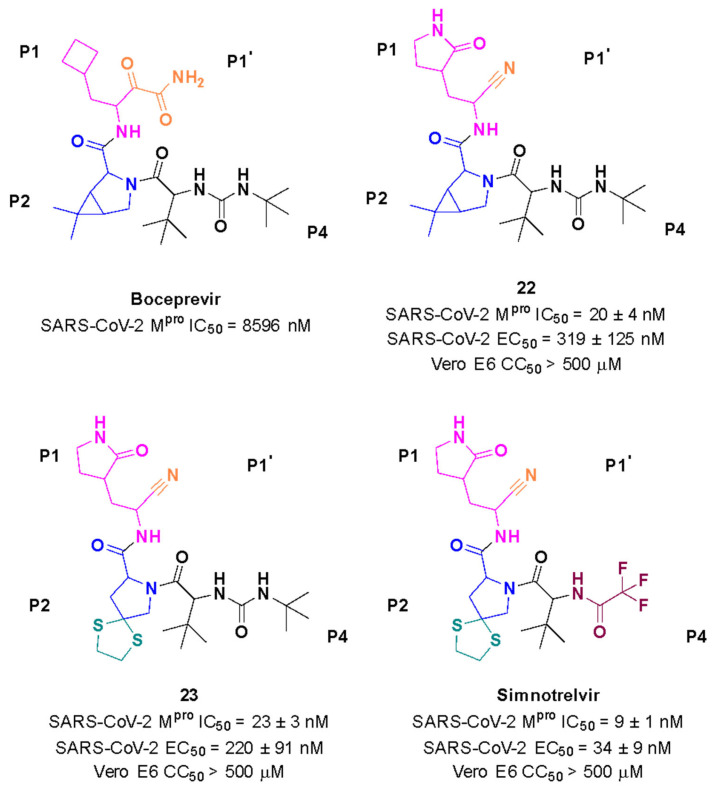
Rational design of simnotrelvir based on boceprevir structure.

Starting from **boceprevir’s** structure, known as an inhibitor of the HCV serine protease, Jiang and colleagues (2023) proposed molecular modifications to enhance the potency of inhibition of the SARS-CoV-2 M^pro^, resulting in the development of simnotrelvir (Figure 27) [146,149]. As demonstrated with other inhibitors, such as **GC-3769**, **lufotrelvir**, and **nirmatrelvir** (Figure 9), the presence of a five-membered lactam ring at the P1 position is favorable for interaction with the M^pro^ enzyme [99,101,104]. Therefore, the first modification involved substituting the cyclobutyl group at this position in **boceprevir**. Subsequently, three different warheads were tested at the P1’ position: an aldehyde, a nitrile, and an α,β-unsaturated ketone. Among these, compound **22**, containing a nitrile group, demonstrated the best IC_50_ in the protein inhibition assays, with values of 20 ± 4 nM (Figure 27). Crystal structure analyses revealed that the dimethyl cyclopropyl-proline group at the P2 position of **boceprevir** did not fully occupy the M^pro^ S2 subsite. As a result, it was replaced with various groups, including phenoxy-proline, phenyl-proline, phenylthiol-proline, dithiaspiro-proline, and bicyclo-proline. The substitution with dithiaspiro-proline resulted in compound **23** showing the best IC_50_ value in assays against the enzyme (IC_50_ 23 ± 3 nM). The final optimization involved modifications at the P4 position, where the substitution of the tert-butylamide group with trifluoroacetyl, 1-fluorocyclopropane-1-acetyl, methylsulfonyl, and cyclopropanesulfonyl groups was evaluated. The best results were achieved with the trifluoroacetyl group at this position, which resulted in **simnotrelvir**, with an IC_50_ of 9 ± 1 nM against the M^pro^ and an EC_50_ of 34 ± 9 nM in assays with Vero E6 cells infected with SARS-CoV-2. Additionally, this compound exhibited no cytotoxicity, with a CC_50_ greater than 500 µM (Figure 27) [146].

Preclinical and phase 1 studies with simnotrelvir (**NCT05339646**) demonstrated that only 22.2% of patients experienced adverse effects from therapy with **simnotrelvir** (150–3000 mg) or **simnotrelvir/ritonavir** (250–1200 mg). These adverse effects included diarrhea, abdominal pain, electrocardiogram abnormalities, and increased serum creatinine levels. Furthermore, the analysis revealed that the concentration of the drug needed to inhibit 90% (EC_90_) of SARS-CoV-2 replication in vitro, as determined in assays using infected Vero E6 cells, was 750 mg of **simnotrelvir** administered twice daily with 100 mg of **ritonavir** [147]. 

Subsequently, a randomized, double-blind, placebo-controlled phase 1b study (**NCT05369676**) evaluated the combination of 750 mg of **simnotrelvir** with 100 mg of **ritonavir**, as well as 300 mg of simnotrelvir with 100 mg of **ritonavir**, in treating adults with asymptomatic, mild, or moderate COVID-19 infection. The results suggested that the combination of 750 mg of **simnotrelvir** with 100 mg of **ritonavir** was generally well tolerated and could be recommended for further clinical use [150].

In a phase 2/3 study involving 1208 patients (**NCT05506176**), treatment with 750 mg of **simnotrelvir** and 100 mg of ritonavir, administered within 72 h of symptom onset, showed significant improvements compared to the placebo group, evidenced by a reduction in the viral load from baseline by day 5. Furthermore, the adverse events reported in the treatment group were generally mild or moderate [151].

Another phase 1 clinical study (**NCT05475834**), a non-randomized, open-label, single-dose trial of simnotrelvir/ritonavir, is underway to evaluate the mass balance, biotransformation, safety, and tolerability of the drug in healthy adult Chinese males. The results are not available yet [152].

Ongoing studies continue to monitor its long-term safety and effectiveness, as well as potential interactions with other medications. The results presented for simnotrelvir shed light on the potential for a new anti-COVID-19 treatment.

## Figures and Tables

**Table 1:** Number of results of the PubMed search of the terms “(covid19) AND (covalent/non-covalent inhibitor) AND (main protease) OR (3CLpro) OR (papain-like protease)”.

Year	Search Term	
	Covalent	Non-Covalent
**2020**	38	13
**2021**	76	31
**2022**	94	33
**2023**	90	25
**2024**	49	16
**Total**	347	118

**Table 2:** Results of in vitro SARS-CoV-2 M^pro^ inhibitory activity of baicalein and some compounds from series A and B.

Compound	Structure	IC_50_^a^ (μM)	Compound	Structure	IC_50_^a^ (μM)
**Baicalein ^b^**	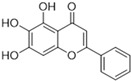	0.966 ± 0.065	**B7**	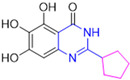	0.539 ± 0.061
**12**	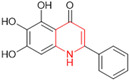	No Inhibition	**B11**	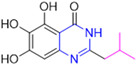	0.385 ± 0.024
**13**	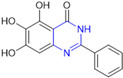	1.372 ± 0.047	**B15**	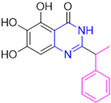	0.174 ± 0.038
**A4**	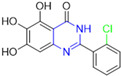	0.435 ± 0.041	**B16**	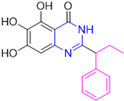	0.210 ± 0.028
**A10**	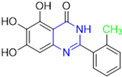	0.365 ± 0.033			

^a^ IC_50_ value refers to the concentration of the compound required to inhibit M^pro^ activity by 50%. ^b^ Baicalein was used as the positive control.

**Table 3:** Results of in vitro SARS-CoV-2 M^pro^ inhibitory activity of baicalein and selected compounds from C and D series.

Compound	Structure	IC_50_^a^ (μM)	Compound	Structure	IC_50_^a^ (μM)
**Baicalein ^b^**	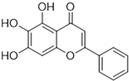	0.966 ± 0.065	**C12**	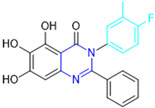	0.117 ± 0.016
**C1**	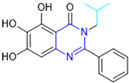	0.124 ± 0.018	**D1**	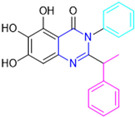	0.477 ± 0.078
**C5**	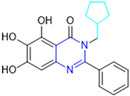	0.124 ± 0.016	**D6**	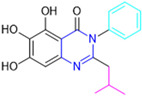	0.107 ± 0.023
**C7**	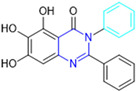	0.083 ± 0.006	**D8**	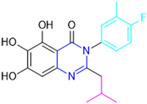	0.100 ± 0.012

^a^ IC50 value refers to the concentration of the compound required to inhibit M^pro^ activity by 50%. ^b^ Baicalein was used as the positive control.

**Table 4:** Physicochemical properties and stability of tested compounds and baicalein.

Compound	MDCK		Kinetic Solubility ^c^ (μM)	PPB ^d^ (%)	CL_int_^e^ (μL/min/mg Protein)	t_1/2_^f^ (min)
	Papp ab ^a^ (cm/s)	ER ^b^				
**Baicalein**	6.07 × 10^−6^	0.34	112.18	0.93	333.05	4.16
**C7**	9.67 × 10^−6^	0.44	179.35	0.70	108.68	12.75
**D6**	7.45 × 10^−6^	0.40	155.98	2.61	68.34	20.28
**D8**	7.83 × 10^−6^	0.41	Not tested	3.17	179.51	7.72

^a^ MDCK: membrane permeability in canine kidney cells (MDCK), with Papp ab (cm/s) being the apparent permeability across MDCK cell monolayers. Permeability categories are as follows (units of 10^−6^ cm/s): low < 1, moderate = 1–10, high > 10. A:B, apical-to-basolateral. B:A, basolateral-to-apical. ^b^ Efflux ratio. ^c^ Kinetic aqueous solubility in PBS (pH = 7.4). ^d^ Human plasma protein binding (PPB), fractions unbound (%) of compounds in plasma. ^e^ Metabolic stability toward human liver microsomes (HLMs) in the presence of NADPH and UDPGA. ^f^ Half-time of the compounds in human liver microsomes (HLMs) in the presence of NADPH and UDPGA.

**Table 5:** Results of evaluation of cytotoxicity and antiviral activity of baicalein and selected compounds in Vero E6 cells infected with SARS-CoV-2.

Compound	EC_50_^a^ (μM)	CC_50_^b^ (μM)
**C7**	1.10 ± 0.12	>50
**D6**	2.87 ± 1.43	>50
**D8**	2.11 ± 1.16	>50
**Baicalein**	5.15 ± 2.46	>50

^a^ EC_50_ is the concentration capable of inhibiting 50% of SARS-CoV-2 replication in Vero E6 cells. ^b^ CC_50_ is the concentration capable of reducing the cell viability by 50%.

**Table 6:** Results of in vitro inhibitory activity of Mei derivatives against SASR-CoV-2 M^pro^.

Compound	Structure	% M^pro^ Inhibition (10 μM)	IC_50_^a^ (μM)	Compound	Structure	%M^pro^ Inhibition (10 μM)	IC_50_ (μM)
**Mei**	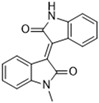	50.6	N.T. ^b^	**Ebselen**	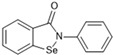	97.5	N.T.
**S14-1**	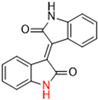	85.3	1.20	**SS14-5**	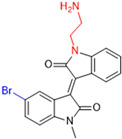	85.8	1.09
**S14-2**	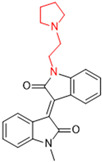	86.5	1.15	**S14-6**	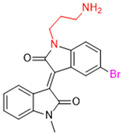	90.5	1.35
**S14-3**	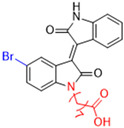	97.8	1.21	**S14-7**	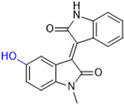	84.2	N.T.
**S14-4**	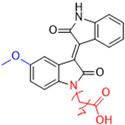	89.1	1.61				

^a^ IC50 value refers to the concentration of the compound required to inhibit Mpro activity by 50%. ^b^ N.T. = not tested.

**Table 7:** The antiviral activity of nirmatrelvir and selected compounds in HeLa-hACE2 cells (MOI 0.03) infected with SARS-CoV-2.

Compound	Structure	EC_50_	
		Spike ^a^	Nucleocapsid ^b^
**Nirmatrelvir**	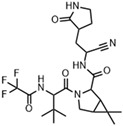	14.12 nM	16.94 nM
**S14-2**	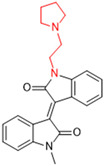	No activity	No activity
**S14-5**	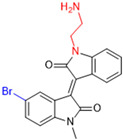	1.76 µM	2.03 µM
**S14-6**	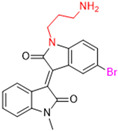	2.74 µM	2.66 µM

^a^ Concentration of the compound capable of reducing the expression of the spike protein by 50%. ^b^ Concentration of the compound capable of reducing the expression of the nucleocapsid protein by 50%. Both proteins’ relative expression levels were quantified by qRT-PCR.

**Table 8:** Physicochemical properties and stability against simulated gastric and intestinal fluids of Mei derivatives and residual percentages in mouse liver microsomes of compounds **S14-6**.

Compound	Water Solubility (mg/mL)	LogP	Residual % ^a^ (Gastric)	Residual % ^b^ (Intestinal)	t_1/2_^c^ (min)	CL_int_^d^ (μL min^−1^mg^−1^)	Residual % ^e^ (Mouse Liver Microsomes)
**S14-2**	17.31	−0.70	95.16	85.26	N.T.	N.T.	N.T.
**S14-5**	3.81	−0.59	95.05	77.98	N.T.	N.T.	N.T.
**S14-6**	2.49	−0.29	95.28	73.64	495	1.4	84

^a^ Residual percentage of compound in simulated gastric fluid at 24 h post-treatment. ^b^ Residual percentage of compound in simulated intestinal fluid at 24 h post-treatment. ^c^ Hepatic microsomal metabolic half-life. ^d^ Clearance. ^e^ Residual percentage of **S14-6** after two hours of incubation in mouse liver microsomes.

**Table 9:** Results of in vitro PL^pro^ inhibitory activity and antiviral activity against SARS-CoV of compounds derived from **7724772**.

Compound	Structure	IC_50_^a^ (µM)	EC_50_^b^ (µM)
**7724772** **(racemate)**	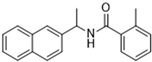	20.1 ± 1.1	NI ^c^
**7724772 (*S*)**	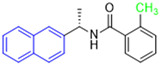	>200	NT ^d^
**7724772 (*R*)**	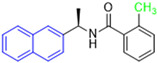	20.1 ± 1.1	NI
**15**	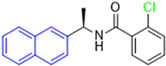	14.5 ± 0.9	NI
**16**	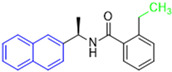	>200	NT
**17**	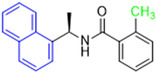	2.3 ± 0.1	10.0 ± 1.2
**18**	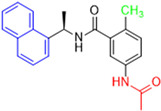	2.6 ± 0.1	13.1 ± 0.7
**19**	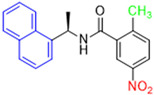	7.3 ± 0.9	NI
**GLR-0617**	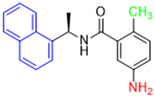	0.6 ± 0.1	14.5 ± 0.8

^a^ IC_50_ is the concentration capable of inhibiting 50% of PL^pro^ activity. ^b^ EC_50_ is the concentration capable of inhibiting 50% of SARS-CoV replication in Vero E6 cells. ^c^ NI = no inhibition. ^d^ NT = not tested.

**Table 10:** Results of in vitro SARS-CoV-2 PL^pro^ assay of positive controls **GLR0617** and **XR8-89** and four most active inhibitors identified.

Compound	Structure	PL^pro^ Inhibition ^a^ (%)	IC_50_^b^ (µM)
**GLR0617**	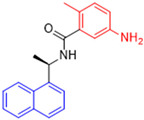	100	0.8 (0.7–1.0)
**XR8-89**	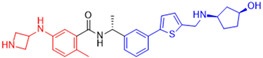	N.T. ^c^	0.64 (0.6–0.7)
**VPC-300141**	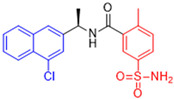	100	5.3 (4.4–6.3)
**VPC-300195**	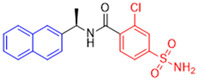	100	4.4 (3.8–5.3)
**VPC-300016**	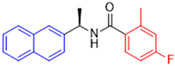	70	8.9 (7.4–10.6)
**VPC-300002**	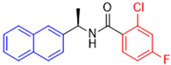	65	18.7 (12.8–27.4)

^a^ PL^pro^ inhibition is the percentage inhibition of the enzyme activity in the screening performed with the compounds at 130 µM. ^b^ IC_50_ is the concentration capable of inhibiting 50% of the PL^pro^ activity. ^c^ N.T.: not tested.

**Table 11:** IC_50_ values of positive controls nirmatrelvir and **XR8-89** and selected compounds from VPC series against replication-competent clone of SARS-CoV-2 Wuhan Hu-1.

Compound	IC_50_ (µM)			
	−CP100356 ^a^ Pre-Incubation time			+CP100356 Pre-Incubation Time
	0 h	1 h	3 h	0 h
**Nirmatrelvir**	9.5	3.1	3.9	2.3
**VPC-300002**	>100	>100	47.1	>100
**VPC-300016**	>100	>100	48.0	>100
**VPC-300141**	>100	>100	42.5	>100
**VPC-300195**	>100	>100	15	17.9
**XR8-89**	>100	>100	40.3	32.0

^a^ **CP100356** is an inhibitor of efflux transporters. In the absence of **CP100356**, the compounds were pre-incubated with cells for 0, 1, or 3 h. In the presence of **CP100356**, the compounds were assessed without being pre-incubated with cells.

**Table 12:** Results of in vitro SARS-CoV-2 PL^pro^ inhibitory activity of compounds **21a–c**.

Compound	Structure	% Inhibition ^a^			IC_50_^b^ (nM)
		5000 nM	500 nM	50 nM	
**Ebselen**	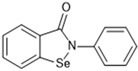	ND ^c^	ND	ND	2688 ± 23.2
**GLR-0617**	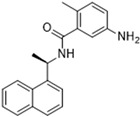	90.9 ± 6.4	ND	ND	1547 ± 14.5
**21a**	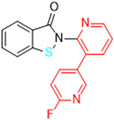	101.6 ± 5.6	96.3 ± 5.8	69.3 ± 4.9	53.9 ± 1.2
**21b**	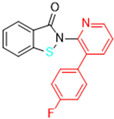	103.1 ± 7.7	90.7 ± 7.3	53.4 ± 5.6	59.9 ± 6.7
**21c**	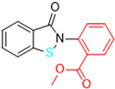	101.5 ± 7.5	90.5 ± 6.1	44.9 ± 8.2	63.1 ± 6.7

^a^ The % inhibition is the average inhibition percentage of the PL^pro^ at 5000 nM, 500 nM, and 50 nM in duplicated tests. ^b^ IC_50_ values for PL^pro^ are averages of three independent assays. All values are expressed as the mean ± SD from triplicate measurements. ^c^ ND = not determined.

**Table 13:** Results of in vitro SARS-CoV-2 M^pro^ inhibitory activity of ebselen and compounds **21a–c**.

Compound	IC_50_^a^ (nM)	Percentage Inhibition at 500 nM ^b^ (%)
**Ebselen (500 nM)**	ND ^c^	43.1 ± 6.1
**21a**	315.8 ± 5.4	ND
**21b**	539.4 ± 6.8	ND
**21c**	438.5 ± 7.6	ND

^a^ IC_50_ values for M^pro^ are averages of three independent assays. ^b^ The percentage inhibition is the average inhibition percentage of the M^pro^ activity at 500 nM in duplicated tests. All values are expressed as the mean ± SD from triplicate measurements. ^c^ ND = not determined.

**Table 14:** The antiviral activity of the controls ebselen and **GLR0617** and compounds **21a** and **21c** in Vero E6 cells infected with SARS-CoV-2.

Compound	EC_50_ (µM)
**Ebselen**	1.6 ± 1.5
**GLR-0617**	21 ± 2
**21a**	7.4 ± 1.9
**21c**	7.4 ± 2.4

**Table 15:** The metabolic stability of compound **21c** in a rat liver homogenate.

Compound	Incubation Time (h)	Remaining Compound ^a^ (%)
**21c**	12	70.6 ± 1.4
	24	53.6 ± 2.1
	48	28.9 ± 2.9

^a^ Remaining compound is the remaining compound content compared with the compound content at 0 h, with this being considered as 100%. All values are expressed as the mean ± SD from triplicate measurements.
